# Increased Glutamate Plus Glutamine in the Right Middle Cingulate in Early Schizophrenia but Not in Bipolar Psychosis: A Whole Brain ^1^H-MRS Study

**DOI:** 10.3389/fpsyt.2021.660850

**Published:** 2021-06-07

**Authors:** Juan R. Bustillo, Elizabeth G. Mayer, Joel Upston, Thomas Jones, Crystal Garcia, Sulaiman Sheriff, Andrew Maudsley, Mauricio Tohen, Charles Gasparovic, Rhoshel Lenroot

**Affiliations:** ^1^Department of Psychiatry and Behavioral Sciences, University of New Mexico, Albuquerque, NM, United States; ^2^Department of Mathematics and Statistics, University of New Mexico, Albuquerque, NM, United States; ^3^Department of Radiology, University of Miami, Miami, FL, United States; ^4^Mind Research Network, Albuquerque, NM, United States

**Keywords:** glutamate, choline, N-acetyl-aspartate, creatine, spectroscopy, psychosis, schizophrenia, bipolar

## Abstract

Proton magnetic resonance spectroscopy (^1^H-MRS) studies have examined glutamatergic abnormalities in schizophrenia and bipolar-I disorders, mostly in single voxels. Though the critical nodes remain unknown, schizophrenia and bipolar-I involve brain networks with broad abnormalities. To provide insight on the biochemical differences that may underlie these networks, the combined glutamine and glutamate signal (Glx) and other metabolites were examined in patients in early psychosis with whole brain ^1^H-MRS imaging (^1^H-MRSI). Data were acquired in young schizophrenia subjects (*N* = 48), bipolar-I subjects (*N* = 21) and healthy controls (*N* = 51). Group contrasts for Glx, as well as for N-acetyl aspartate, choline, myo-inositol and creatine, from all voxels that met spectral quality criteria were analyzed in standardized brain space, followed by cluster-corrected level alpha-value (CCLAV ≤ 0.05) analysis. Schizophrenia subjects had higher Glx in the right middle cingulate gyrus (19 voxels, CCLAV = 0.05) than bipolar-I subjects. Healthy controls had intermediate Glx values, though not significant. Schizophrenia subjects also had higher N-acetyl aspartate (three clusters, left occipital, left frontal, right frontal), choline (two clusters, left and right frontal) and myo-inositol (one cluster, left frontal) than bipolar-I, with healthy controls having intermediate values. These increases were likely accounted for by antipsychotic medication effects in the schizophrenia subgroup for N-acetyl aspartate and choline. Likewise, creatine was increased in two clusters in treated vs. antipsychotic-naïve schizophrenia, supporting a medication effect. Conversely, the increments in Glx in right cingulate were not driven by antipsychotic medication exposure. We conclude that increments in Glx in the cingulate may be critical to the pathophysiology of schizophrenia and are consistent with the NMDA hypo-function model. This model however may be more specific to schizophrenia than to psychosis in general. Postmortem and neuromodulation schizophrenia studies focusing on right cingulate, may provide critical mechanistic and therapeutic advancements, respectively.

## Introduction

The N-methyl-D-aspartate receptor (NMDAR) hypo-function model of psychosis originated from pharmacological studies documenting the emergence of positive and negative symptoms as well as cognitive deficits in healthy volunteers exposed acutely to the NMDAR blocker ketamine (1). Also, acute systemic NMDAR blockers in the awake rat lead to an increase in frontal extracellular glutamate (1). Consistent with these findings, a single ketamine infusion in healthy controls (HC) results in an increase in the combined glutamate and glutamine signal (Glx) (2) in medial frontal cortex, as measured with proton magnetic resonance spectroscopy (^1^H-MRS). This paradoxical increase in glutamate release with NMDAR blockers has been postulated to result from higher sensitivity of NMDAR receptors in GABAergic interneurons than in pyramidal neurons, leading to disinhibition of pyramidal neurons (1).

Schizophrenia studies using single-voxel ^1^H-MRS suggest glutamate increases in “basal ganglia” (3) though more recent studies at 7T in early antipsychotic-treated psychosis found prefrontal glutamate reductions (4, 5). Furthermore, never-treated schizophrenia patients had no glutamate differences relative to healthy control subjects in dorsal anterior cingulate (6), but increases have been documented in the dorsal striatum in this population (7). Like schizophrenia, bipolar-I is a chronic, neurodevelopmental disorder, often presents with psychotic symptoms, and is commonly treated with antipsychotic medications (8). Several ^1^H-MRS studies have reported increased glutamatergic measures in bipolar-I (9) [but see (10)]. Hence, the literature on ^1^H-MRS-measured glutamate in psychotic disorders is far from clear, with region of interest, medication status, chronicity and co-morbidity as possible confounders. To partially address some of these issues, a few direct comparisons between bipolar-I and schizophrenia have been implemented. Two of these used single-voxel ^1^H-MRS and did not ascertain a history of psychosis in the bipolar-I sample (11, 12). Our previous study used single slice spectroscopic imaging and involved mainly chronic patients, all with history of psychosis (13). However, none of these three studies found glutamatergic differences between the two clinical groups.

Schizophrenia and bipolar-I are disorders that likely involve distributed brain networks and subtle global brain volume reductions (generally greater in schizophrenia than bipolar-I) have been repeatedly documented (14). Three-dimensional proton MR spectroscopic imaging (3D ^1^H-MRSI) enables measurement in much larger brain regions, thereby reducing the bias of voxel selection intrinsic to single-voxel studies. Using 3D ^1^H-MRSI with a short echo time (TE) and a voxel-wide approach, we recently reported reduced Glx in the left superior temporal gyrus (STG) in early schizophrenia vs. healthy control subjects as well as more widespread creatine increases in antipsychotic treated schizophrenia (15).

Here we report the use of the same imaging tool to compare Glx and other metabolites of interest in early schizophrenia vs. early bipolar-I disorder with psychotic features. Healthy control subjects (HC) were also examined to assist in interpretation. We include the schizophrenia (*N* = 36) and HC (*N* = 29) subjects' data previously reported plus an additional sample of schizophrenia, HC, and bipolar-I subjects. Consistent with our previous findings we expected Glx reductions in STG in schizophrenia (15) but not in bipolar-I. We also hypothesized higher creatine in antipsychotic-treated vs. antipsychotic-naïve schizophrenia (15).

## Methods and Materials

### Subjects

Schizophrenia (Sz) and bipolar-I (BP-I) subjects were recruited from the University of New Mexico Hospitals (UNMH). Inclusion criteria were: (1) Schizophrenia, schizophreniform, schizoaffective or bipolar-I disorder with psychotic features made using the SCID-DSM-5; (2) Age between 16 and 40. Exclusion criteria were diagnosis of current substance use disorder (except for nicotine) or a neurological disorder. Healthy controls in the same age range were recruited from the community and excluded if they had: (1) any current DSM-5 disorder (SCID-DSM-5 Non-Patient-Version; except for nicotine use); (2) history of neurological disorder; or (3) first-degree relatives with any psychotic disorder. The UNMH Institutional Review Board approved the study and all subjects provided written informed consent and were reimbursed for their participation. Forty-eight Sz, 21 BP-I, and 51 HCs participated.

### Clinical and Neuropsychological Assessments

Cognitive function was assessed in clinical and HC groups using the Measurement-and-Treatment-Research-to-Improve-Cognition-in-Schizophrenia (MATRICS) battery (11). Patients were assessed for psychopathology with the Positive-and Negative-Syndrome-Scale (16), the Young-Mania-Rating-Scale (17), and the Calgary-Depression-Scale (18). Patients were also evaluated for extra-pyramidal side-effects with the Abnormal-Involuntary-Movements-Scale (19), Simpson-Angus-Scale for Parkinsonism (20), and the Barnes-Akathisia-Scale (21). These assessments were completed within 1 week of the scan acquisition.

### Magnetic Resonance Studies

#### Acquisition

Subjects underwent an MR study at 3T using a Siemens TIM Trio scanner as previously described (15). 3D ^1^H-MRSI was acquired using an echo-planar-spectroscopic-imaging (EPSI) sequence with the following parameters: TE = 17.6 ms, TR = 1,551 ms, TR (H_2_O) = 511 ms, non-selective lipid inversion nulling with TI = 198 ms, FOV = 280 × 280 × 180 mm, voxel size of 5.6 × 5.6 × 10 mm, echo train length of 1,000 points, bandwidth of 2,500 Hz, reduced k-space sampling (acceleration factor = 0.7), a nominal voxel volume of 0.313 cm^3^ and acquisition time of 17 min (15). EPSI included a water reference measurement that was interleaved with the metabolite signal acquisition. The data processing pipeline has been fully described (15) and is briefly summarized below.

#### Reconstruction and Registration

EPSI reconstruction and analysis was carried out using the MIDAS package (22). Processing included corrections for B_0_ shifts, generation of white-matter, gray-matter, and CSF tissue segmentation maps using FMRIB Software Library (FSL) FAST (https://fsl.fmrib.ox.ac.uk/fsl/fslwiki/), lipid k-space extrapolation and linear registration of the T_1_-weighted MR images to the EPSI images. Metabolite maps were interpolated to 64 × 64 × 32 points (voxel size 4.375 × 4.375 × 5.625 mm). Following spatial smoothing the effective voxel volume was 1.55 cm^3^. EPSI water reference measurement was spatially registered to the MPRAGE T_1_-weighted image.

#### Spectral Fitting

**Figure 1:**
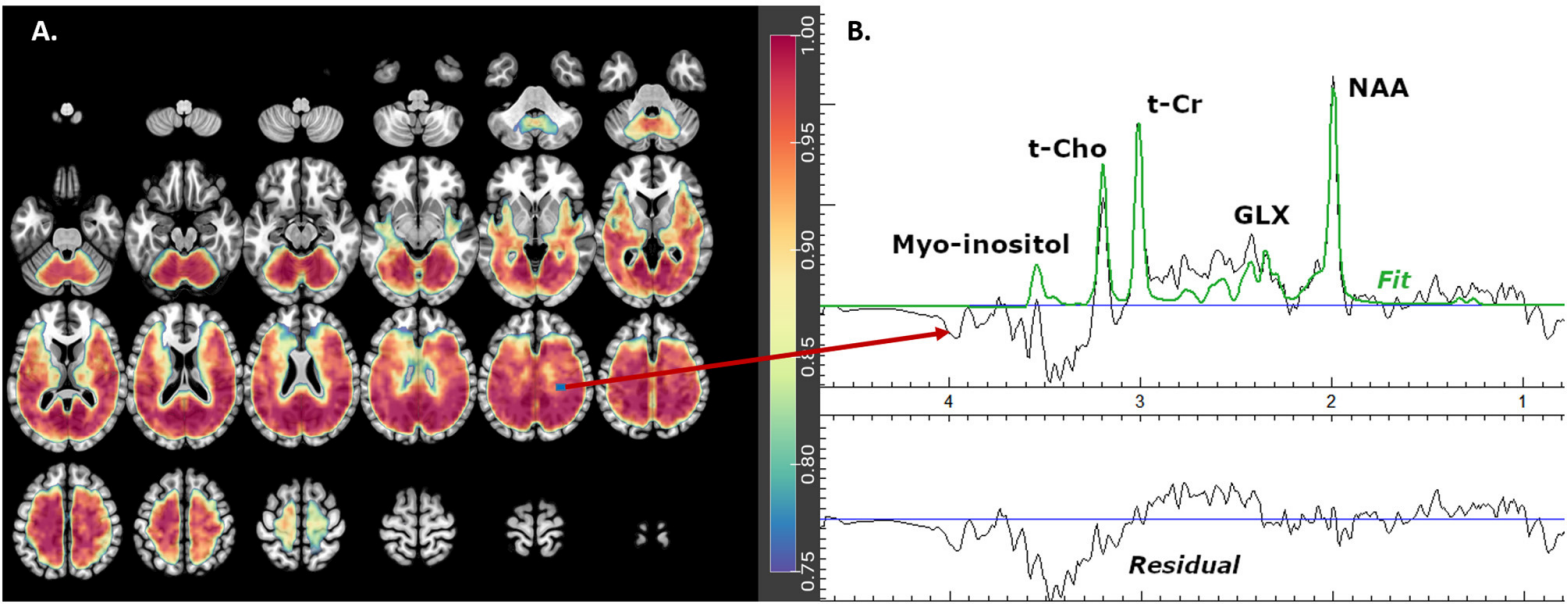
Glx mask showing **(A)** axial brain slices with useful spectral coverage (color grading from blue- [0.75 coverage in each group] to red- [1.0 coverage in each group]) and **(B)** example of one fitted spectrum. Glx is glutamate plus glutamine, NAA is N-acetyl-aspartate compounds, t-Cho is choline, glycerol-phospho-choline, and phosphocholine and t-Cr is creatine plus phospho-creatine.

The fitted metabolite values from the spectra were estimated using MIDAS and spectral analysis was carried out using the FITT2 program for: Glx, N-acetyl-aspartate compounds (NAA), total creatine (creatine and phosphocreatine, denoted t-Cr), total choline (choline, glycerol-phosphocholine, and phosphocholine, denoted t-Cho), and myo-inositol (Figure 1). Individual metabolite maps were normalized and corrected for any bias field variations using the signal from the water reference measurement corrected for tissue water density from literature values. These values were not corrected for relaxation rates and represent institutional units.

#### Warping and Quality Filtering

The metabolite maps were exported from MIDAS and warped using Statistical Parametric Mapping-8 (SPM8; https://www.fil.ion.ucl.ac.uk/spm/software/spm8/) to the Montreal Neurological Institute (MNI) space (http://brainmap.org/training/BrettTransform.html), keeping the interpolated voxel size, so that group analysis could be performed. Subsequently, to correct for partial volume effects, the metabolite maps values are divided by 1-*f*_*CSF*_ at the voxel level. For spectral quality, the metabolite maps were filtered according to three criteria: overall line widths = 2–12 Hz; specific spectral fits for each metabolite of Cramér–Rao lower bound (CRLB) = 1–20%; and *f*_*CSF*_ ≤ 0.3 (these were labeled *best spectra*). Spectra that failed to meet these criteria were divided into two groups, *poor* and *intermediate* spectra. *Intermediate spectra* met the following criteria: linewidths >1 and less than or =16; CRLB = 1–99%; *f*_*CSF*_ ≤ 0.3; and at least 18 of the 26 nearest neighboring voxels were *best spectra. Intermediate spectra* were considered to have some potential useful spectral information and hence their metabolite values were kept. All others were considered *poor spectra* and their metabolite values were discarded.

#### Metabolite Group Mask

These masks (one for each metabolite) included only the *best* and *intermediate spectra* voxels that were present in ≥ 75% of subjects in each diagnostic group (Figure 1). The masks were then smoothed using a spatially stationary Gaussian filter with a kernel width of 10mm in SPM12 to minimize potential spatial warping errors.

#### Imputation

Since up to 25% of subjects in each diagnostic group may not have a particular voxel value in the metabolite group mask after filtering out voxels according to the above quality criteria, the metabolite values for the *poor spectra* voxels were imputed using the diagnostic group (Sz, BP-I, HC) mean concentration for that voxel. This allowed us to preserve the maximum number of voxels possible for the analysis, without adding variability to the populations' metabolite concentrations. Imputations only accounted for an average of between 2 and 7% of the total spectra in each metabolite group mask.

### Statistical Analyses

The principal analyses examined diagnostic group (Sz vs. BP-I) differences separately for each of the five metabolites of interest. Adjustments were made for age at a subject level and for gray matter proportion [GM/(GM+WM)] at the voxel level. These two factors have large effects on metabolite's variability (23). Analysis of Functional NeuroImages [AFNI's; (24)] *3dttest*++*, 3dclust* and *3dClustSim* packages were used because they support both subject and voxel-level covariates (24). *3dClustSim* computed cluster-size thresholds using 10,000 simulated noise-only *t*-tests for a more accurate spatial autocorrelation function of the noise. It estimated the probability of false positive clusters and then corrected the voxel threshold to reduce the likelihood of false positive clusters. With *3dttest*++, voxel-wise Student t-tests were implemented for all voxels that fell within the supported quality group mask. The resulting maps were clustered using *3dclust* following three criteria: (1) each voxel differed between the groups with *p* ≤ 0.001; (2) group differences for each voxel in the cluster were in the same direction; and (3) voxels had faces-touching. The corrected clustering thresholds from *ClustSim* were compared to the number of voxels clustered in *3dclust* to insure the cluster met a corrected cluster-level alpha-value (CCLAV) of ≤ 0.05. Lastly, Kendall's *Tau* (τ) tested correlations between the gray matter proportion-weighted average concentrations for clusters that differed between groups and clinical and cognitive assessments.

## Results

### Demographic and Clinical Variables

Subjects were similarly aged, with no significant differences in gender, race, parental socioeconomic status (SES), cardiovascular risk, or history of substance use disorders, except for cannabis and current smoking (Table 1). Sz and BP-I had greater history of cannabis use disorder (*p* < 0.001) and current smoking (*p* = 0.03) as well as worse personal SES (*p* < 0.001) and MATRICS overall t-scores (*p* < 0.001) than HCs. The psychosis groups were similarly young (Sz age = 22.4, BP-I = 22.2) and early in the illness (Sz onset = 20.2, BP-I = 21.1). The two groups did not differ in any demographic measures, substance use history, positive or depressive symptoms, proportion of antipsychotic-naïve subjects or antipsychotic dose. Sz subjects had greater negative (*p* = 0.009) and lower manic symptoms (*p* < 0.001) than BP-I subjects.

**Table 1:** Demographic and clinical characteristics.

	**Healthy controls (*N =* 51)**	**Schizophrenia (*N =* 48)**	**Bipolar-I (*N =* 21)**
(Mean ± SD or %)			
Age (years)	23.7 ± 4.2	22.4 ± 3.9	22.2 ± 3.1
Gender (male/female)	57/43%	71/29%	57/43%
Race (Caucasian/Native/Other)	80/8/12%	85/4/11%	95/5/0%
SES^a^	4.1 ± 1.4^*^	6.2 ± 1.4	5.8 ± 1.3
Familial SES	3.6 ± 1.7	4.2 ± 1.8	3.7 ± 1.7
Vascular risk score^b^	0.02 ± 0.14	0.06 ± 0.2	0.19 ± 0.5
MATRICS-overall T score	48.2 ± 7.1^*^	31.50 ± 11.7	34.7 ± 11.9
Smoker (yes/no)	2/98%^*^	10/90%	19/81%
Alcohol (yes/no)	5/95%	12/86%	14/86%
Cannabis (yes/no)	0/100%^*^	52/48%	57/43%
Stimulant (yes/no)	0/100%	4/96%	5/95%
Opioid (yes/no)	0/100%	2/98%	10/90%
Phencyclidine (yes/no)	0/100%	4/96%	10/90%
Hallucinogens (yes/no)	0/100%	0/100%	0/100%
Sedative (yes/no)	0/100%	0/100%	0/100%
Inhalants (yes/no)	0/100%	0/100%	0/100%
Psychosis onset (years)	-	20.2 ± 4.4	21.1 ± 4.2
Positive symptoms	-	16.0 ± 5.0	14.5 ± 6.6
Negative symptoms	-	16.3 ± 5.2	12.6 ± 5.3^∧^
Manic symptoms	-	2.2 ± 3.8	9.4 ± 11.1^∧^
Depressive symptoms	-	2.8 ± 3.9	3.7 ± 3.5
Parkinsonism	-	8.1 ± 0.4	8.2 ± 0.9
Akathisia	-	0.5 ± 1.0	0.5 ± 1.6
Tardive dyskinesia	-	7.0 ± 0.0	7.0 ± 0.0
Antipsychotic use (yes/no)	-	62/38%	60/40%
Antipsychotic dose (mg)^c^	-	6.3 ± 7.0	4.7 ± 5.9%

a *SES is Socioeconomic Status*.

b *Vascular risk score, 0–4 (score of 1 each for cardiac illness, hypertension, dyslipidemia and diabetes)*.

c *Antipsychotic dose, as olanzapine equivalents (25)*.

*Smoker refers to current use. Alcohol, cannabis, stimulant, opioid, phencyclidine, hallucinogens, sedative, inhalants, refer to past substance use disorder per SCID-5*.

* *p ≤ 0.05 for healthy controls vs. schizophrenia or bipolar-I*.

∧ *p ≤ 0.05 for schizophrenia vs. bipolar-I*.

### Effect of Diagnosis: Sz vs. BP-I Group Differences in Neurometabolites

#### Glutamate ± Glutamine

**Figure 2:**
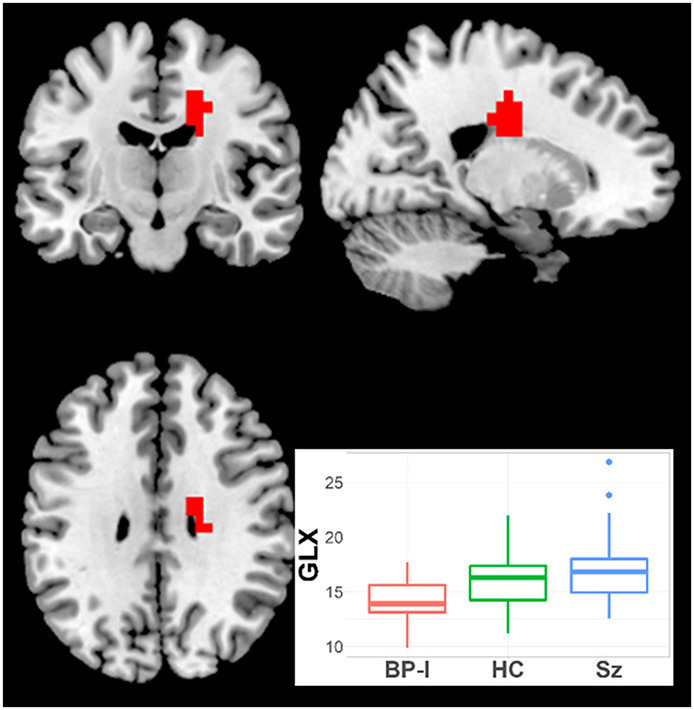
Increased Glx in Schizophrenia (Sz) vs. Bipolar-I (BP) (healthy controls [HC] intermediate) in one cluster (19 voxels), CCLAV = 0.05, including the right cingulate gyrus (60.3%). Boxplot shows weighted-average Glx cluster concentrations in institutional units.

One cluster (19 voxels) had higher Glx in Sz vs. BP-I's (CCLAV = 0.05; mainly in right middle cingulate gyrus (Figure 2, Supplementary Table 1). In this cluster, the HC group had values intermediate, but not significantly different from the clinical groups. Because BP-I subjects had slightly higher Glx CRLB than Sz (Supplementary Table 3), we co-varied for CRLB in the AFNI analysis. The significant Glx cluster (still Sz > BP-I) completely overlapped with the original 19 voxel cluster, but it increased in size to 44 voxels. Finally, within Sz, the antipsychotic-naïve subgroup tended to have higher Glx than the treated patients (though not significantly); this pattern was not present in the BP group (Supplementary Figure 1). The hypothesized left STG Glx reduction was not observed in Sz relative to BP-I or in Sz relative to HCs with CCLAV ≤ 0.05. When a more lenient statistical test focusing on the left STG cluster previously reported (15) was further explored, only 3/19 voxels had lower Glx (*p* < 0.01) in Sz vs. HC.

#### N-acetyl Aspartate

**Figure 3:**
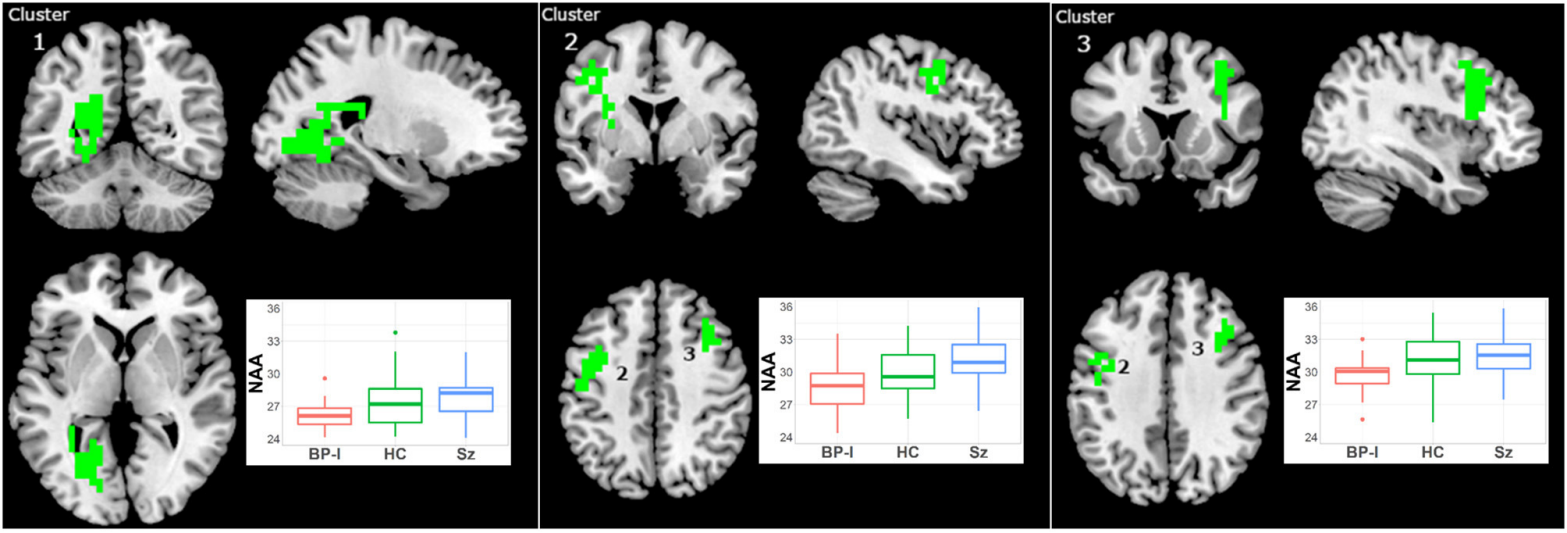
Increased NAA in Schizophrenia (Sz) vs. Bipolar-I (BP) (healthy controls [HC] intermediate) in three clusters: (1) 114 voxels, CCLAV < 0.01, including the left lingual gyrus (29.9%) and left posterior cingulate (13.0%). (2) 61 voxels, CCLAV < 0.01, including the left precentral gyrus (34.7%%) and left middle frontal gyrus (22.1%). (3) 42 voxels, CCLAV = 0.05, including the right middle frontal gyrus (60.6%) and right precentral gyrus (10.2%). Boxplots shows weighted-average NAA cluster concentrations in institutional units.

NAA was higher in Sz vs. BP-I in three clusters (Figure 3, Supplementary Table 1): (1) left occipital (114 voxels; CCLAV <0.01); (2) left frontal (61 voxels; CCLAV ≤ 0.01); and (3) right frontal (42 voxels; CCLAV = 0.05). The HC group had values intermediate (not significant) to those of the clinical groups. Because in the first cluster BP-I subjects had slightly higher NAA CRLB than Sz (Supplementary Table 3), we co-varied for CRLB. The significant NAA cluster 1 (still Sz > BP-I) completely overlapped with the original 114 voxel cluster, but it increased in size to 274 voxels. In all three clusters, for each of the Sz and the BP-I groups, the treated subjects had numerically higher NAA values than antipsychotic-naïve subjects (Supplementary Figures 2a–c).

#### Total Choline

**Figure 4:**
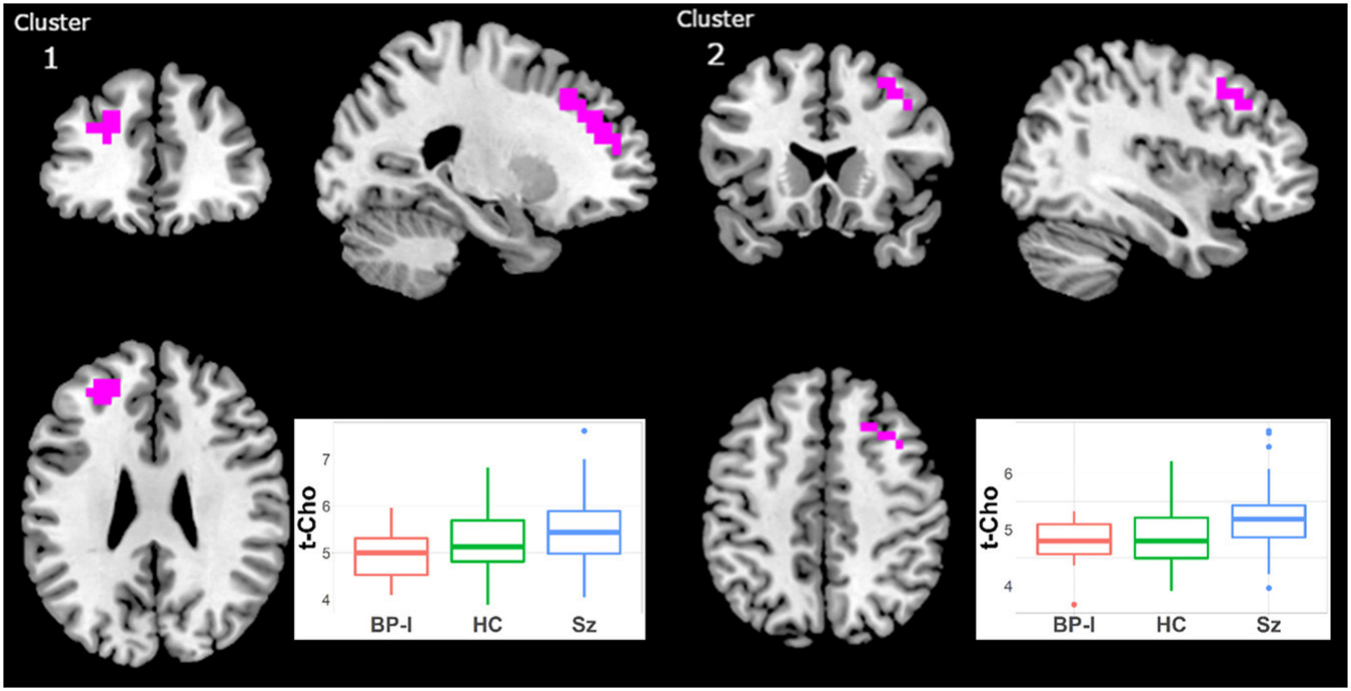
Increased t-Cho in Schizophrenia (Sz) vs. Bipolar-I (BP) (healthy controls [HC] intermediate) in two clusters: (1) 40 voxels, CCLAV = 0.02, including the left superior frontal gyrus (47.3%) and left middle frontal gyrus (39.2%). (2) 29 voxels, CCLAV = 0.03, including the right middle frontal gyrus (47.3%) and the right superior frontal gyrus (14.6%). Boxplots shows weighted-average t-Cho cluster concentrations in institutional units.

t-Cho was higher in Sz than in BP-I subjects in two clusters (Figure 4, Supplementary Table 1): (1) left superior mid-frontal (40 voxels; CCLAV = 0.02); and (2) right superior mid-frontal (29 voxels; CCLAV = 0.03). The HC group had values intermediate (not significant), to those of the clinical groups. In both clusters, for each the Sz and the BP-I groups, the treated subjects had numerically higher t-Cho values than antipsychotic-naïve subjects (Supplementary Figures 3a,b).

#### Myo-Inositol

**Figure 5:**
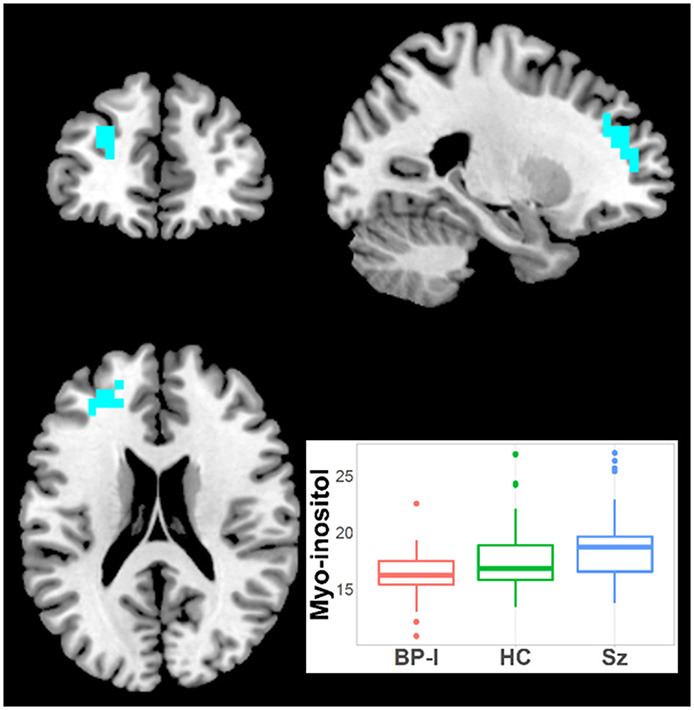
Increased inositol in Schizophrenia (Sz) vs. Bipolar-I (BP) (healthy controls [HC] intermediate) in one cluster (19 voxels), CCLAV = 0.05, including the left superior frontal gyrus (65.7%) and the left middle frontal gyrus (23.1%). Boxplot shows weighted-average inositol cluster concentrations in institutional units.

Myo-inositol was higher in Sz vs. BP-I subjects in one left superior frontal cluster (27 voxels; CCLAV = 0.03; Figure 5). The HCs had intermediate (not significant) metabolite values to those in Sz and BP-I. Amongst the BP-I group, medicated subjects tended to have numerically higher myo-inositol than naïve subjects (Supplementary Figure 4). Finally, there were no differences between Sz and BP-I in t-Cr.

### Antipsychotic Medication Effects: Antipsychotic-Naïve vs. Treated Patients

#### Total-Creatine

**Figure 6:**
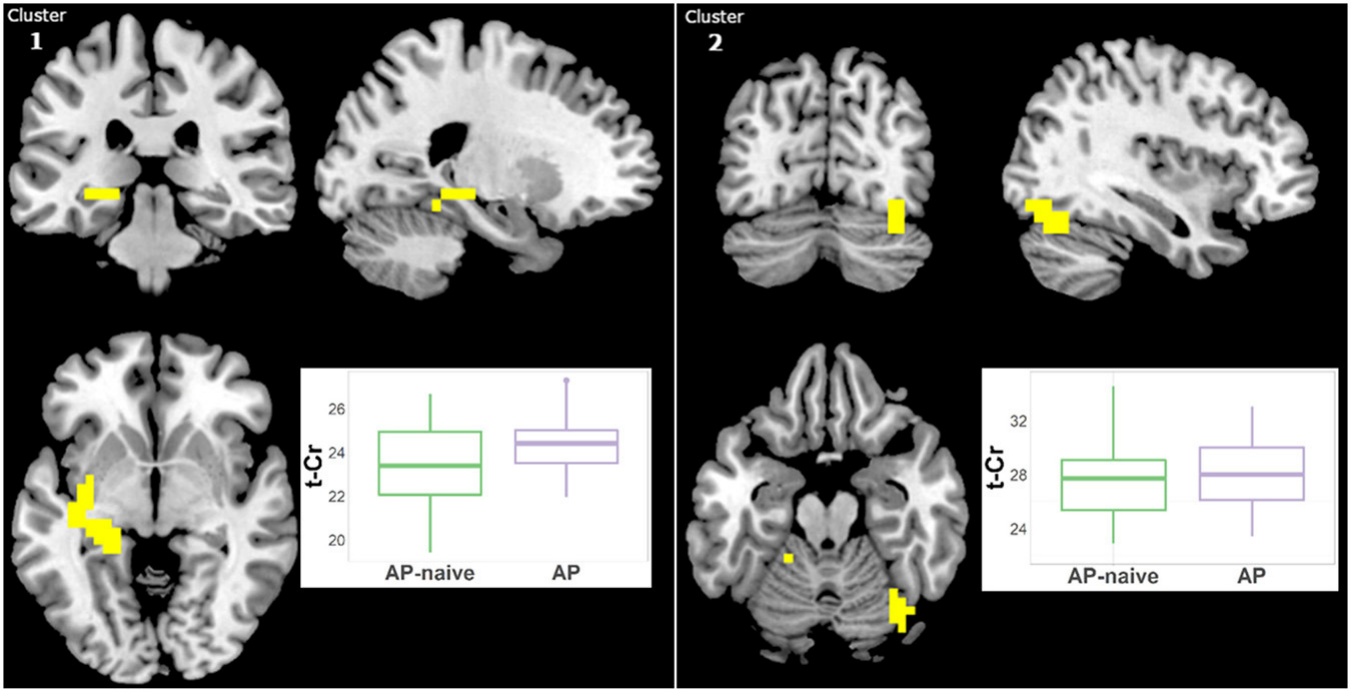
Increased t-Cr in antipsychotic treated (AP) vs. antipsychotic naïve (AP-naive) Schizophrenia in two clusters: (1) 34 voxels (CCLAV = 0.02), including the left parahippocampal gyrus (25.0%) and left culmen (10.5%). (2) 27 voxels, CCLAV = 0.03, including the right declive (51.9%) and the right fusiform gyrus (16.5%). Boxplots show weighted-average t-Cr cluster concentrations in institutional units.

Because we previously detected higher t-Cr in treated Sz relative to HC in three clusters (15) but not in antipsychotic-naïve Sz vs. HCs, we examined in our largest patient sample (i.e., Sz) the effect of antipsychotic medication. Hence, we compared the treated (*N* = 29) and naïve (*N* = 19) Sz subgroups. t-Cr was higher in two clusters in treated patients (Figure 6, Supplementary Table 2): (1) left Para hippocampal (34 voxels; CCLAV = 0.02); and (2) right occipital (27 voxels; CCLAV = 0.03). There were no significant differences between the naïve and treated BP-I subgroups, but the samples were small (*N* = 8 and *N* = 13, respectively).

#### Other Neurometabolites

**Figure 7:**
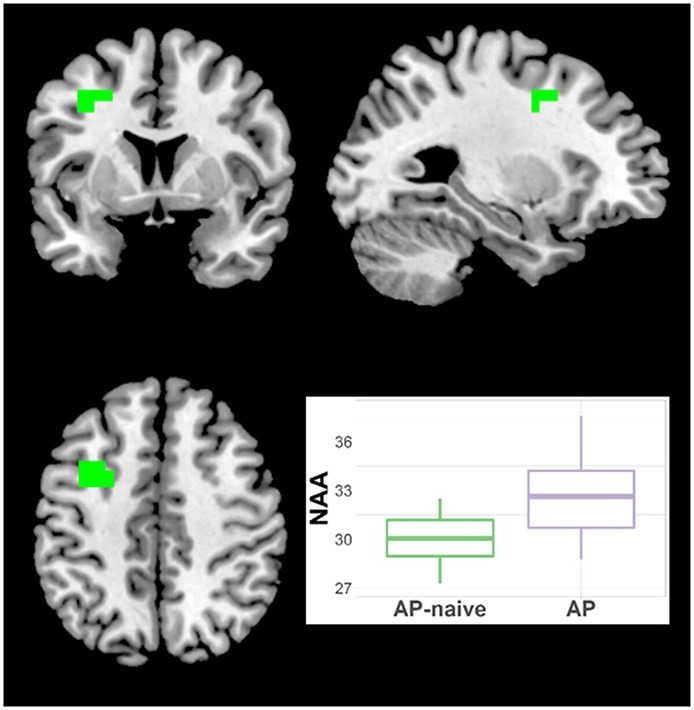
Increased NAA in antipsychotic treated (AP) vs. antipsychotic naïve (AP-naive) Schizophrenia in one cluster: 21 voxels (CCLAV = 0.04), including the left middle frontal gyrus (61.5%%) and left precentral (15.7%). Boxplot shows weighted-average NAA cluster concentrations in institutional units.

**Figure 8:**
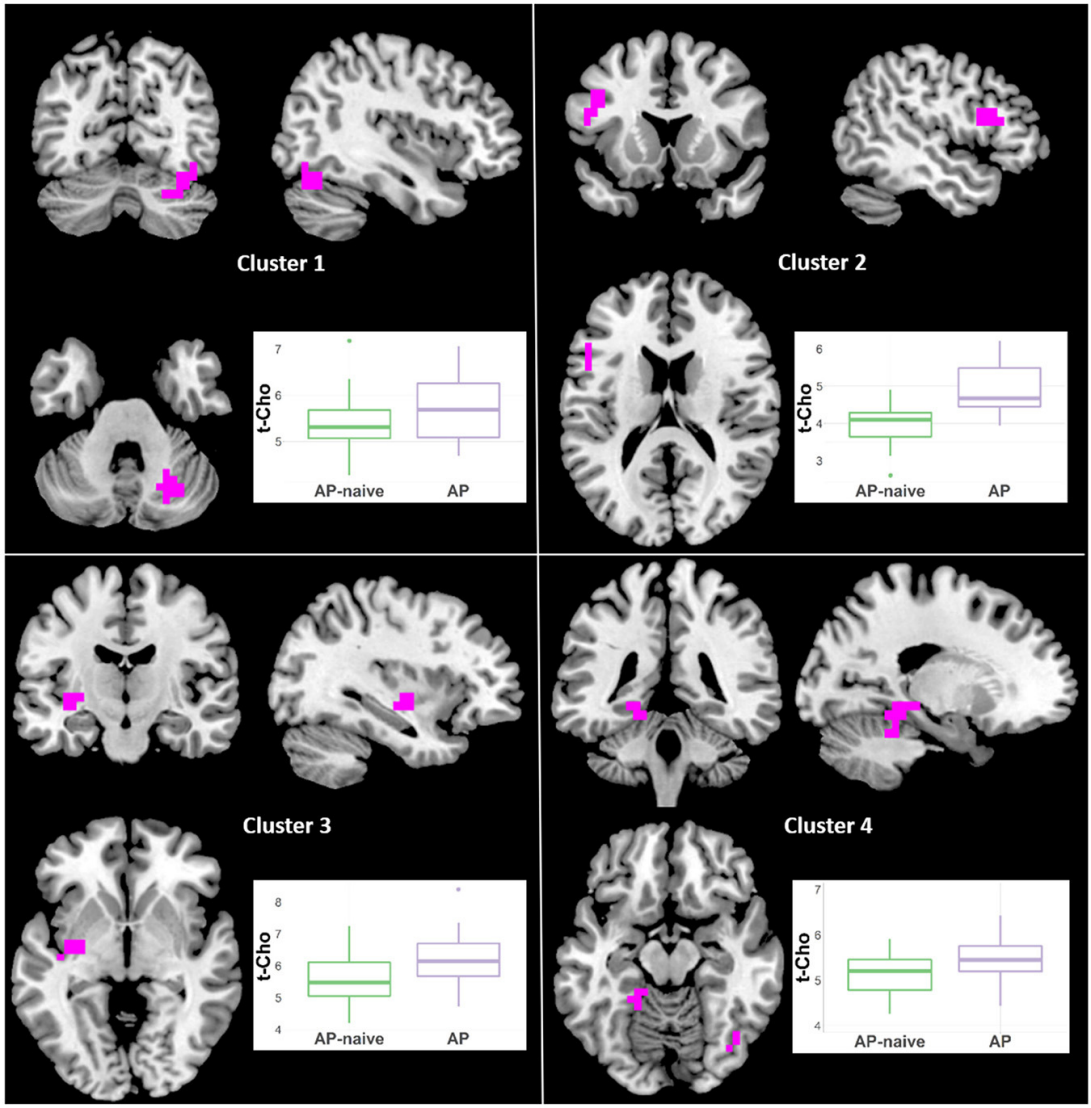
Increased t-Cho in antipsychotic treated (AP) vs. antipsychotic naïve (AP-naive) Schizophrenia in four clusters: (1) 28 voxels; (CCLAV = 0.02), including the right declive (35.6%), right pyramis (19.6%), right tuber (16.3%), and right uvula (11%). (2) 23 voxels; (CCLAV = 0.03), including the left middle frontal gyrus (49.7%), and left inferior frontal gyrus (40%). (3) 18 voxels; (CCLAV = 0.03), including the left insula (21.7%), left claustrum (7.4%) and left lentiform nucleus (7.3%). (4) 16 voxels; (CCLAV = 0.04), including the left parahippocampal gyrus (46.9%), and the left culmen (38.4%).

Because the diagnostic group comparisons suggested effects of antipsychotic medication for Glx, NAA and t-Cho, we compared the Sz naïve and treated subgroups. For NAA there were increments in treated vs. naïve in one left middle frontal cluster (21 voxels; CCLAV = 0.05; Figure 7, Supplementary Table 2). There was higher t-Cho in treated vs. naïve groups in four clusters: (1) right cerebellar cluster (28 voxels; CCLAV = 0.02); (2) left middle frontal cluster (23 voxels; CCLAV = 0.03; (3) left insula cluster (18 voxels; CCLAV = 0.03; and (4) left Para hippocampal cluster (16 voxels; CCLAV = 0.04; Figure 8, Supplementary Table 2). There were no significant group differences for Glx. There were no significant differences between the naïve and treated BP-I subgroups.

#### Symptom and Cognitive Relationships

We examined the correlations between symptoms and cognition with the weighted-average neurochemical concentrations in the clusters that differed between Sz and BP-I (Glx, NAA, t-Cho or myo-inositol; see Supplementary Figures 5–13). Both t-Cho clusters correlated positively with negative symptom severity: (1) left superior mid-frontal (τ = 0.26, *p* = 0.002); and (2) right superior mid-frontal (τ = 0.26, *p* = 0.002). They also correlated negatively with the MATRICS overall t-score: (1) left superior mid-frontal (τ = −0.24, *p* = 0.01); and (2) right superior mid-frontal (τ = −0.24, *p* = 0.01). The one myo-inositol left superior frontal cluster positively correlated with negative symptoms (τ = 0.2, *p* = 0.02). Finally, the three NAA clusters correlated positively with negative symptom scores: (1) left occipital (τ = 0.23, *p* = 0.007); (2) left frontal (τ = 0.21, *p* = 0.01); and (3) right frontal (τ = 0.2, *p* = 0.01). The left frontal NAA cluster correlated negatively with MATRICS overall t-score (τ = −0.23, *p* = 0.01). There were no relationships with positive, manic or depressive symptoms.

## Discussion

To our knowledge this is the first study to compare Glx as well as the other more commonly measured neurometabolites in early schizophrenia and BP-I with psychotic features with a voxel-wise whole brain spectroscopic imaging approach. Several metabolites were higher in Sz subjects compared to BP-I subjects across various regions. Glx was higher in the right middle cingulate, NAA was higher in the left occipital and bilateral frontal areas of cortex, t-Cho was higher in bilateral frontal cortices and myo-inositol was higher in the left frontal regions. These differences were not due to demographic, clinical or spectral quality metrics (see Supplementary Tables 3, 4). The HC group had intermediate values of these metabolites (though not significantly different) relative to the clinical groups, supporting the view that the two early-psychosis groups may have opposite neurometabolic abnormalities: increases in Sz and reductions in BP-I. Additionally we demonstrated effects of antipsychotic treatment in t-Cr in the Sz group, as previously suggested (15). Likewise, the left frontal increases in NAA and t-Cho at least in part appear to be related to antipsychotic treatment. However, the elevated Glx in the right cingulate in Sz appears to be accounted for mainly by the antipsychotic-naïve subjects. Contrary to our hypothesis, Glx in Sz was not lower in the left STG relative to the other groups.

Three ^1^H-MRS studies have directly compared chronically-ill Sz and BP-I subjects at magnetic fields >1.5T (3T and 4T). Two used single-voxel ^1^H-MRS and also did not restrict their sample to BP-I with psychosis (11, 12). In a previous study we used single slice spectroscopic imaging and only included BP-I with history of psychosis (13). None of these three studies found significant glutamatergic differences between Sz and BP-I. Atagun et al. (11) did find higher t-Cr and inositol in the left STG in Sz vs. BP-I. We found some age-related differences with higher NAA and inositol in younger Sz (<40) as well as higher NAA in older Sz relative to older BP-I (13). These results are somewhat consistent with the higher NAA and myo-inositol in early Sz relative to BP-I in the current study.

Increased glutamate, glutamine, and Glx (glutamate + glutamine) in the “basal ganglia” has been reported in one schizophrenia meta-analysis (3). This is consistent with the NMDAR hypo-function pharmacological model of psychosis: acute ketamine induces increased extracellular frontal glutamate in rodents and of ^1^H-MRS glutamatergic measures in healthy humans (1). Though concentrations of glutamate and glutamine measured with ^1^H-MRS do not directly assesses synaptic glutamate turnover, it has been proposed that the acute increment in extracellular glutamate in rodents measured with micro-dialysis reflects synaptic function (1). In humans, the largest published ^1^H-MRS ketamine challenge study reported increased Glx in anterior cingulate in a single-voxel (2). Hence, though less specific, Glx may be a more sensitive ^1^H-MRS index for the paradoxical effect of higher glutamate observed in acute NMDAR inhibition by ketamine. In the present study, the one cluster with increased Glx in Sz involved a more posterior aspect of the right dorsal cingulate. We note, however, that with the whole-brain ^1^H-MRSI approach we used, we may have been less able to detect group differences in the ventral and anterior cingulate, due to worse spectral quality in these regions (Figure 1). Likewise, the head of the caudate and the anterior thalamus, regions where glutamate increments have been described in antipsychotic-naïve schizophrenia (3), were mostly inaccessible with our approach.

The failure to confirm our previously reported Glx reduction in left STG in early Sz (15) was not due to differences in spectral quality between the original (CRLB = 7.58 ± 1.24) and the added Sz samples (CRLB = 7.07 ± 1.33). Also, the voxel coverage was the same in both groups (16 voxels over left STG). The two samples did differ in the number of antipsychotic-naïve Sz subjects: 57% in the original and 12% in the added samples (Supplementary Table 5). A histogram of z-scores of the examined voxels (Supplementary Figure 14) shows that the data from the current sample had a greater spread of Sz vs. HC differences, with the added subjects having z-scores around zero (i.e., no group effect). However, examination of the histogram z-scores did not support a greater effect in antipsychotic-naïve vs. HC than in treated Sz vs. HC (Supplementary Figure 14).

Results of previous spectroscopic studies examining glutamatergic metabolites are likely to have been affected or limited by antipsychotic medication, chronicity, and the brain region examined. More recent single-voxel studies at 7T in treated early schizophrenia have reported reduced glutamate in anterior cingulate (4, 5), suggesting an effect of antipsychotic medication. Consistently, antipsychotic-naïve schizophrenia patients had increased dorsal striatal glutamate, which normalized with prospective risperidone treatment (7). However, a recent study in antipsychotic-naïve schizophrenia failed to detect any differences in anterior cingulate glutamate (6). Furthermore, two 7T studies of the anterior cingulate in chronically-ill patients found glutamate reductions (26, 27). Hence, the inconsistencies in the results of glutamate studies in schizophrenia may be in part due to differences in stage of illness, medication effects and regions of interest with the single-voxel approach. Clearly, psychotic disorders involve multiple distributed brain networks (14) hence, examining glutamatergic measures with an unbiased voxel-wise approach offers advantages for future studies. Though antipsychotic medications may lower glutamate in some regions (7), glutamatergic dysfunction intrinsic to the illness is supported by the report of several glutamate-related risk-conferring genes in in the largest GWAS study in schizophrenia (28). Finally, in terms of diagnostic specificity our results suggest that glutamatergic abnormalities may differ fundamentally in bipolar-I and schizophrenia, with elevations in schizophrenia, consistent with a primary NMDAR hypofunction in GABA-ergic interneurons, and reductions in bipolar-I, suggestive of a different glutamatergic deficit, perhaps of NMDAR dysfunction in pyramidal neurons. However, the Glx differences were not related to symptom severity, like manic or negative symptoms, which suggests a trait effect of diagnosis.

Higher NAA, t-Cho and myo-inositol in Sz relative to BP-I were unexpected findings in the current study. The majority of the literature on schizophrenia reports lower NAA mainly in “basal ganglia” and frontal lobe (29). However, a recent meta-analysis of cross-sectional studies reported progressive, stage of illness-related NAA reductions: only in hippocampus in high-risk subjects; in frontal and thalamic regions in early schizophrenia; and more widespread reductions (frontal, hippocampal, temporal, thalamic and parietal regions) in chronically-ill patients (30). This is consistent with progressive brain volume reductions from multiple longitudinal MRI studies (31). In BP-I, lower NAA in “basal ganglia” has been reported (29). There is no consistent evidence of alterations in t-Cho or t-Cr in either disorder (29). The findings on myo-inositol have been more sparse, but lower concentrations in the medial frontal region was reported in a meta-analysis in schizophrenia (32). However, the great majority of the analyzed data came from single-voxel studies (29, 32). None of the studies included involved whole brain with voxel-wise analyses.

The literature on antipsychotic medication effects on ^1^H-MRS measures was recently expanded by a meta-analysis of single-voxel longitudinal schizophrenia studies before and after treatment (33). Frontal Glx was found to be reduced and thalamic NAA increased by medication. In this meta-analysis no changes in inositol were found and t-Cho and t-Cr were not examined. However, one study reported increased medial temporal t-Cr and inositol in treated vs. anti-psychotic-naïve schizophrenia, suggesting a medication effect for these metabolites (34).

Antipsychotics clearly have acute metabolic effects with increased subcortical and reduced cortical metabolism (35, 36). In addition, these agents can induce striatal volume expansion in humans as well as cortical volume reductions as early as 12 weeks after treatment (37). In monkeys, antipsychotics induce global gray matter volume reductions (38) with a 14% glial reduction and 10% increase in neuronal density (39). Hence, because NAA is mainly found in neurons (40) [though also reported in immature oligodendrocytes (41)], it is possible that increased neuronal density causes a relative increase in NAA tissue concentration, which could account for the higher NAA findings in the treated early schizophrenia subgroup of the present study. The consistent more widespread NAA reductions reported later in the illness (30) suggest an effect of chronicity, perhaps related to loss of neuropil without gliosis as described in the postmortem literature (42).

We did confirm increased t-Cr in antipsychotic-treated vs. naïve Sz subgroups consistent with our previous report (15). The higher t-Cr with treatment suggests an effect on energy metabolism. A reduction in the forward rate constant of creatine kinase, the enzyme that converts creatine to phosphocreatine, was reported in antipsychotic-treated schizophrenia (43). Hence, the increase in t-Cr may represent an adaptation to the cortical metabolism-lowering effects of antipsychotics (35, 36), by increasing the total pool of creatine available for energetic transfer. Though alterations in t-Cho have not been consistently reported in single-voxel studies of schizophrenia (29), long-term exposure to antipsychotics in monkeys has been reported to result in increased frontal glial density (44) and reduced parietal glial density (39). Because the t-Cho signal represents cell membrane turnover (40), we speculate that the finding of higher t-Cho only in treated vs. naïve schizophrenia patients may suggest an adaptive glial response to antipsychotics in frontal cortex bilaterally.

The associations between some of the metabolite clusters that differed between Sz and BP-I and symptoms and cognition (for the whole clinical sample) were not predicted and do not survive multiple comparison correction. However, some intriguing patterns emerged. Mainly in bilateral frontal regions, higher t-Cho correlated with worse negative symptoms and more impaired cognition. Not surprisingly, negative symptoms and cognition were negatively correlated (τ = −0.38, *p* < 0.001). This may suggest that increased frontal glial density (as reflected in higher t-Cho) may result in worsened negative symptoms and cognition in psychosis. However, the similar association between bilateral frontal NAA clusters and negative symptoms is harder to interpret as higher NAA is usually described as an index of improved neuronal viability (40).

This study has several strengths. 3D EPSI allowed examination of most of the brain, including numerous gray and white matter regions known to be affected in schizophrenia and bipolar-I (14). The use of AFNI permitted a voxel-wise approach with false-positive correction, as is standard with other neuroimaging modalities. AFNI also allowed introduction of co-variates at the voxel level, a critical issue in ^1^H-MRS analyses since the partial volume tissue effects are substantial (23). However, some limitations should be acknowledged. First, the spectral resolution with the current sequence did not allow reliable discrimination of glutamate from glutamine, hence Glx results are presented. Second, though whole brain ^1^H-MRSI was acquired, greater magnetic field inhomogeneity precluded examination of more ventral regions, such as orbitofrontal and inferior temporal areas. Third, our sample of 21 BP-I is small relative to the other two groups. Finally, as this was a case-control design, mechanistic interpretations are inherently limited.

In summary, this study of early psychosis using voxel-wise examination with 3D ^1^H-MRSI revealed an increase in glutamate metabolism (Glx) in the right middle cingulate in schizophrenia but not in bipolar-I. This increase did not appear to be related to antipsychotic treatment or due to other common confounders, such as substance use or chronicity. Also, the metabolite levels of the HC group, were intermediate between those of that Sz and BP-I groups. This suggests that Sz and BP-I may have abnormalities in metabolism that alter glutamate in opposite directions. In other regions explored in the current study, NAA, t-Cho, and myo-inositol were also higher in Sz. This differs in particular with the broad schizophrenia literature of reduced NAA in mainly chronically-ill schizophrenia populations examined with single voxels. However, in our sample medication status appears to account statistically for the higher NAA and t-Cho. Likewise, higher t-Cr appears clearly related to antipsychotic treatment. Replication of these findings would support mechanistic postmortem investigations and therapeutic neuromodulation studies of the right cingulate in schizophrenia.

## Data Availability Statement

The raw data supporting the conclusions of this article will be made available by the authors, without undue reservation.

## Ethics Statement

The studies involving human participants were reviewed and approved by Human Research Programs Office, UNM Health Sciences Center. The patients/participants provided their written informed consent to participate in this study.

## Author Contributions

JB and RL were involved in the design, acquisition, analysis, interpretation of data for the work, drafting, and revising of the manuscript. EM, JU, TJ, and CGar were involved in the analysis, interpretation of data for the work, drafting, and revising of the manuscript. SS, AM, MT, and CGas were involved in the interpretation of data for the work, drafting, and revising of the manuscript. All authors contributed to the article and approved the submitted version.

## Conflict of Interest

The authors declare that the research was conducted in the absence of any commercial or financial relationships that could be construed as a potential conflict of interest.

## References

[1] Moghaddam B Javitt D. From revolution to evolution: the glutamate hypothesis of schizophrenia and its implication for treatment. Neuropsychopharmacology. (2012) 37:4–15. 10.1038/npp.2011.181 21956446 PMC3238069

[2] Javitt DC Carter CS Krystal JH Kantrowitz JT Girgis RR Kegeles LS. Utility of imaging-based biomarkers for glutamate-targeted drug development in psychotic disorders: a randomized clinical trial. JAMA Psychiatry. (2018) 75:11–9. 10.1001/jamapsychiatry.2017.3572 29167877 PMC5833531

[3] Merritt K Egerton A Kempton MJ Taylor MJ McGuire PK. Nature of glutamate alterations in schizophrenia: a meta-analysis of proton magnetic resonance spectroscopy studies. JAMA Psychiatry. (2016) 73:665–74. 10.1001/jamapsychiatry.2016.0442 27304221

[4] Wang AM Pradhan S Coughlin JM Trivedi A DuBois SL Crawford JL. Assessing brain metabolism with 7-t proton magnetic resonance spectroscopy in patients with first-episode psychosis. JAMA Psychiatry. (2019) 76:314–23. 10.1001/jamapsychiatry.2018.3637 30624573 PMC6439827

[5] Reid MA Salibi N White DM Gawne TJ Denney TS Lahti AC. 7T proton magnetic resonance spectroscopy of the anterior cingulate cortex in first-episode schizophrenia. Schizophr Bull. (2019) 45:180–9. 10.1093/schbul/sbx190 29385594 PMC6293230

[6] Li J Ren H He Y Li Z Ma X Yuan L. Anterior cingulate cortex glutamate levels are related to response to initial antipsychotic treatment in drug-naive first-episode Schizophrenia Patients. Front Psychiatry. (2020) 11:553269. 10.3389/fpsyt.2020.553269 33192666 PMC7644538

[7] de la Fuente-Sandoval C Leon-Ortiz P Azcarraga M Stephano S Favila R Diaz-Galvis L. Glutamate levels in the associative striatum before and after 4 weeks of antipsychotic treatment in first-episode psychosis: a longitudinal proton magnetic resonance spectroscopy study. JAMA Psychiatry. (2013) 70:1057–66. 10.1001/jamapsychiatry.2013.289 23966023 PMC3790718

[8] Birur B Kraguljac NV Shelton RC Lahti AC. Brain structure, function, and neurochemistry in schizophrenia and bipolar disorder-a systematic review of the magnetic resonance neuroimaging literature. NPJ Schizophrenia. (2017) 3:15. 10.1038/s41537-017-0013-9 28560261 PMC5441538

[9] Gigante AD Bond DJ Lafer B Lam RW Young LT Yatham LN. Brain glutamate levels measured by magnetic resonance spectroscopy in patients with bipolar disorder: a meta-analysis. Bipolar Disord. (2012) 14:478–87. 10.1111/j.1399-5618.2012.01033.x 22834460

[10] Cao B Stanley JA Selvaraj S Mwangi B Passos IC Zunta-Soares GB. Evidence of altered membrane phospholipid metabolism in the anterior cingulate cortex and striatum of patients with bipolar disorder I: A multi-voxel (1)H MRS study. J Psychiatr Res. (2016) 81:48–55. 10.1016/j.jpsychires.2016.06.006 27376506

[11] Atagun MI Sikoglu EM Can SS Karakas-Ugurlu G Ulusoy-Kaymak S Caykoylu A. Investigation of Heschl's gyrus and planum temporale in patients with schizophrenia and bipolar disorder: a proton magnetic resonance spectroscopy study. Schizophr Res. (2015) 161:202–9. 10.1016/j.schres.2014.11.012 25480359 PMC4308441

[12] Ongur D Jensen JE Prescot AP Stork C Lundy M Cohen BM. Abnormal glutamatergic neurotransmission and neuronal-glial interactions in acute mania. Biol Psychiatry. (2008) 64:718–26. 10.1016/j.biopsych.2008.05.014 18602089 PMC2577764

[13] Bustillo JR Jones T Qualls C Chavez L Lin D Lenroot RK. Proton magnetic resonance spectroscopic imaging of gray and white matter in bipolar-I and schizophrenia. J Affect Disord. (2019) 246:745–53. 10.1016/j.jad.2018.12.064 30623820

[14] Thompson PM Jahanshad N Ching CRK Salminen LE Thomopoulos SI Bright J. ENIGMA and global neuroscience: a decade of large-scale studies of the brain in health and disease across more than 40 countries. Transl Psychiatry. (2020) 10:100. 10.1016/j.biopsych.2020.02.167 32198361 PMC7083923

[15] Bustillo JR Upston J Mayer G Jones T Maudsley AA Gasparovic C. Glutamatergic hypo-function in the left superior and middle temporal gyri in early schizophrenia: a data-driven three-dimensional proton spectroscopic imaging study. Neuropsychopharmacology. (2020) 45:1851–9. 10.1038/s41386-020-0707-y 32403117 PMC7608301

[16] Kay SR Fiszbein A Opler LA. The positive and negative syndrome scale (PANSS) for schizophrenia. Schizophr Bull. (1987) 13:261–76. 10.1093/schbul/13.2.261 3616518

[17] Young RC Biggs JT Ziegler VE Meyer DA. A rating scale for mania: reliability, validity and sensitivity. Br J Psychiatry. (1978) 133:429–35. 10.1192/bjp.133.5.429 728692

[18] Addington D AJ Maticka-Tyndale E. Assessing depression in schizophrenia: the calgary depression scale. Br J Psychiatry Suppl. (1993) 1993:39–44. 10.1192/S0007125000292581 8110442

[19] Schooler NR Kane JM. Research diagnoses for tardive dyskinesia. Arch Gen Psychiatry. (1982) 39:486–7. 10.1001/archpsyc.1982.04290040080014 6121550

[20] Simpson GM Angus JW. A rating scale for extrapyramidal side effects. Acta Psychiatr Scand Suppl. (1970) 212:11–9. 10.1111/j.1600-0447.1970.tb02066.x 4917967

[21] Barnes TR. A rating scale for drug-induced akathisia. Br J Psychiatry. (1989) 154:672–6. 10.1192/bjp.154.5.672 2574607

[22] Maudsley AA Darkazanli A Alger JR Hall LO Schuff N Studholme C. Comprehensive processing, display and analysis for in vivo MR spectroscopic imaging. NMR Biomed. (2006) 19:492–503. 10.1002/nbm.1025 16763967 PMC2673915

[23] Maudsley AA Domenig C Govind V Darkazanli A Studholme C Arheart K. Mapping of brain metabolite distributions by volumetric proton MR spectroscopic imaging (MRSI). Magn Reson Med. (2009) 61:548–59. 10.1002/mrm.21875 19111009 PMC2724718

[24] Cox RW. AFNI: software for analysis and visualization of functional magnetic resonance neuroimages. Comput Biomed Res. (1996) 29:162–73. 10.1006/cbmr.1996.0014 8812068

[25] Gardner DM Murphy AL O'Donnell H Centorrino F Baldessarini RJ. International consensus study of antipsychotic dosing. Am J Psychiatry. (2010) 167:686–93. 10.1176/appi.ajp.2009.09060802 20360319

[26] Brandt AS Unschuld PG Pradhan S Lim IA Churchill G Harris AD. Age-related changes in anterior cingulate cortex glutamate in schizophrenia: A (1)H MRS Study at 7 Tesla. Schizophr Res. (2016) 172:101–5. 10.1016/j.schres.2016.02.017 26925800 PMC4821673

[27] Kumar J Liddle EB Fernandes CC Palaniyappan L Hall EL Robson SE. Glutathione and glutamate in schizophrenia: a 7T MRS study. Mol Psychiatry. (2020) 25:873–82. 10.1038/s41380-018-0104-7 29934548 PMC7156342

[28] Ripke S Neale BM Corvin A Walters JTR Farh KH Holmans PA. Biological insights from 108 schizophrenia-associated genetic loci. Nature. (2014) 511:421–+. 10.1038/nature13595 25056061 PMC4112379

[29] Kraguljac NV Reid M White D Jones R den Hollander J Lowman D. Neurometabolites in schizophrenia and bipolar disorder - a systematic review and meta-analysis. Psychiatry Res. (2012) 203:111–25. 10.1016/j.pscychresns.2012.02.003 22981426 PMC3466386

[30] Whitehurst TS Osugo M Townsend L Shatalina E Vava R Onwordi EC. Proton magnetic resonance spectroscopy of n-acetyl aspartate in chronic schizophrenia, first episode of psychosis and high-risk of psychosis: a systematic review and meta-analysis. Neurosci Biobehav Rev. (2020) 119:255–67. 10.1016/j.neubiorev.2020.10.001 33068555

[31] Olabi B Ellison-Wright I McIntosh AM Wood SJ Bullmore E Lawrie SM. Are there progressive brain changes in schizophrenia? A meta-analysis of structural magnetic resonance imaging studies. Biol Psychiatry. (2011) 70:88–96. 10.1016/j.biopsych.2011.01.032 21457946

[32] Das TK Dey A Sabesan P Javadzadeh A Theberge J Radua J. Putative Astroglial Dysfunction in Schizophrenia: A Meta-Analysis of (1)H-MRS Studies of Medial Prefrontal Myo-Inositol. Front Psychiatry. (2018) 9:438. 10.3389/fpsyt.2018.00438 30298023 PMC6160540

[33] Kubota M Moriguchi S Takahata K Nakajima S Horita N. Treatment effects on neurometabolite levels in schizophrenia: a systematic review and meta-analysis of proton magnetic resonance spectroscopy studies. Schizophr Res. (2020) 222:122–32. 10.1016/j.schres.2020.03.069 32505446

[34] Wood SJ Berger GE Wellard RM Proffitt T McConchie M Velakoulis D. A 1H-MRS investigation of the medial temporal lobe in antipsychotic-naive and early-treated first episode psychosis. Schizophr Res. (2008) 102:163–70. 10.1016/j.schres.2008.03.012 18456460

[35] Lahti AC Holcomb HH Weiler MA Medoff DR Tamminga CA. Functional effects of antipsychotic drugs: comparing clozapine with haloperidol. Biol Psychiatry. (2003) 53:601–8. 10.1016/S0006-3223(02)01602-5 12679238

[36] Lahti AC Weiler MA Medoff DR Tamminga CA Holcomb HH. Functional effects of single dose first- and second-generation antipsychotic administration in subjects with schizophrenia. Psychiatry Res. (2005) 139:19–30. 10.1016/j.pscychresns.2005.02.006 15950442

[37] Lieberman JA Tollefson GD Charles C Zipursky R Sharma T Kahn RS. Antipsychotic drug effects on brain morphology in first-episode psychosis. Arch Gen Psychiatry. (2005) 62:361–70. 10.1001/archpsyc.62.4.361 15809403

[38] Dorph-Petersen KA Pierri JN Perel JM Sun Z Sampson AR Lewis DA. The influence of chronic exposure to antipsychotic medications on brain size before and after tissue fixation: a comparison of haloperidol and olanzapine in macaque monkeys. Neuropsychopharmacology. (2005) 30:1649–61. 10.1038/sj.npp.1300710 15756305

[39] Konopaske GT Dorph-Petersen KA Pierri JN Wu Q Sampson AR Lewis DA. Effect of chronic exposure to antipsychotic medication on cell numbers in the parietal cortex of macaque monkeys. Neuropsychopharmacology. (2007) 32:1216–23. 10.1038/sj.npp.1301233 17063154

[40] Rae CD. A guide to the metabolic pathways and function of metabolites observed in human brain 1H magnetic resonance spectra. Neurochem Res. (2014) 39:1–36. 10.1007/s11064-013-1199-5 24258018

[41] Urenjak J Williams SR Gadian DG Noble M. Specific expression of N-acetylaspartate in neurons, oligodendrocyte-type-2 astrocyte progenitors, and immature oligodendrocytes in vitro. J Neurochem. (1992) 59:55–61. 10.1111/j.1471-4159.1992.tb08875.x 1613513

[42] Harrison PJ. The neuropathology of schizophrenia. A critical review of the data and their interpretation. Brain J Neurol. (1999) 122 :593–624. 10.1093/brain/122.4.593 10219775

[43] Du F Cooper AJ Thida T Sehovic S Lukas SE Cohen BM. In vivo evidence for cerebral bioenergetic abnormalities in schizophrenia measured using 31P magnetization transfer spectroscopy. JAMA Psychiatry. (2014) 71:19–27. 10.1001/jamapsychiatry.2013.2287 24196348 PMC7461723

[44] Selemon LD Lidow MS Goldman-Rakic PS. Increased volume and glial density in primate prefrontal cortex associated with chronic antipsychotic drug exposure. Biol Psychiatry. (1999) 46:161–72. 10.1016/S0006-3223(99)00113-4 10418690

