# Supplementary material for: Increased Glutamate Plus Glutamine in the Right Middle Cingulate in Early Schizophrenia but Not in Bipolar Psychosis: A Whole Brain 1H-MRS Study
Source: Front Psychiatry. 2021 Jun 7;12:660850. doi: 10.3389/fpsyt.2021.660850 (PMC8215955; doi:10.3389/fpsyt.2021.660850)
Supplement: Supplementary file 1 [file Data_Sheet_1.docx]

**Supplementary Figures**

**Figures 1 through 4:** Histograms representing non-significant neurometabolite distribution differences between antipsychotic treated (AP) vs anti-psychotic naïve (AP-naïve) subjects within the bipolar-I (BP-I; top panel) and the schizophrenia (Sz) groups (bottom panel). The number of voxels (y-axis) are part of the significantly different clusters (in Sz vs BP-I) and their Z-score (x-axis) reflect how these vary from the healthy controls’ values (red vertical line). Distributions to the right of the red line suggest AP have numerically higher neurometabolite values than AP-naïve.

**Figure 1. Glx cluster: numerically higher in AP-naïve than AP-treated in Sz.**

**
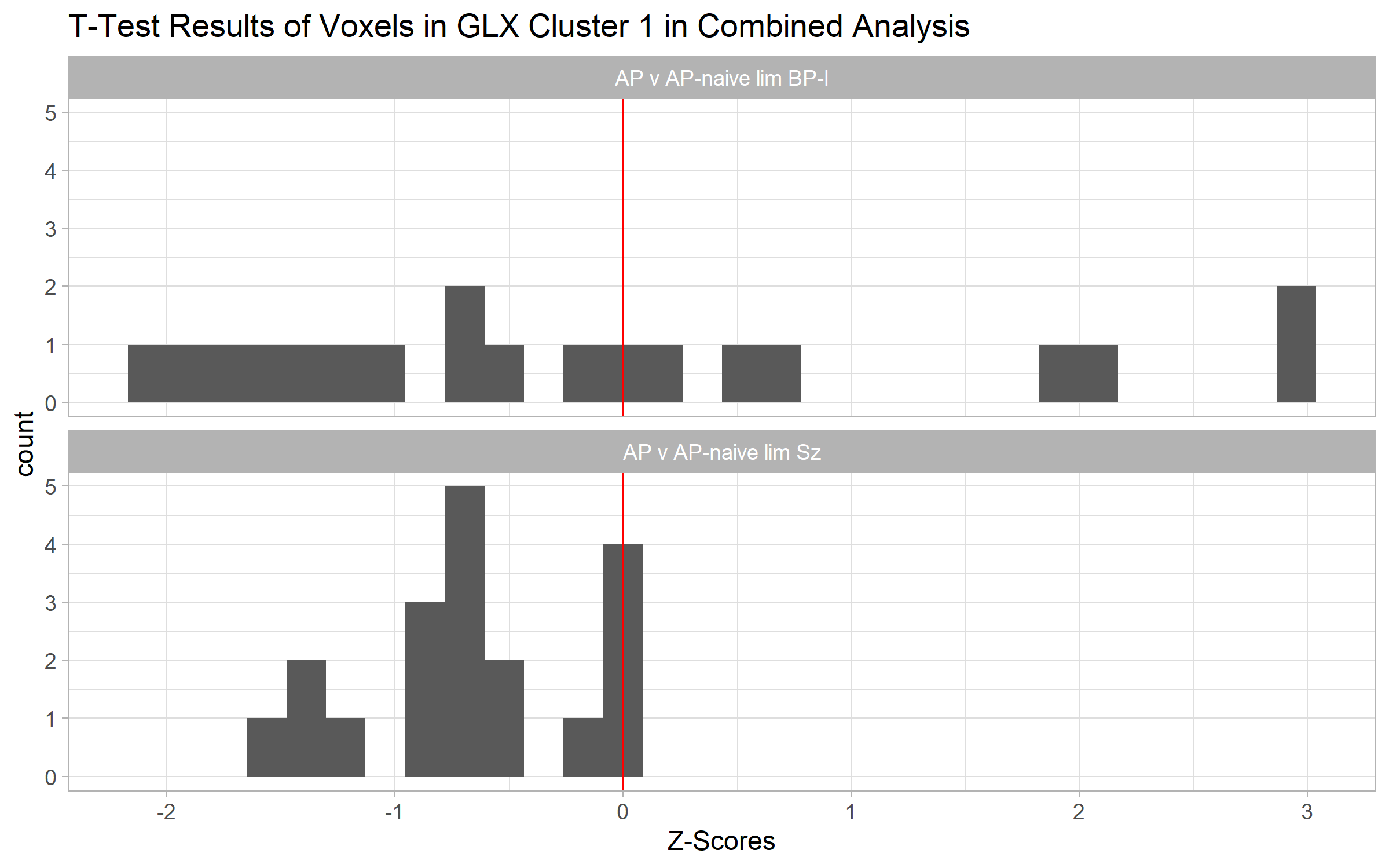
**

**Figure 2a. NAA, cluster 1****: numerically higher in AP-treated than in AP-naïve in Sz and BP-I.**

**
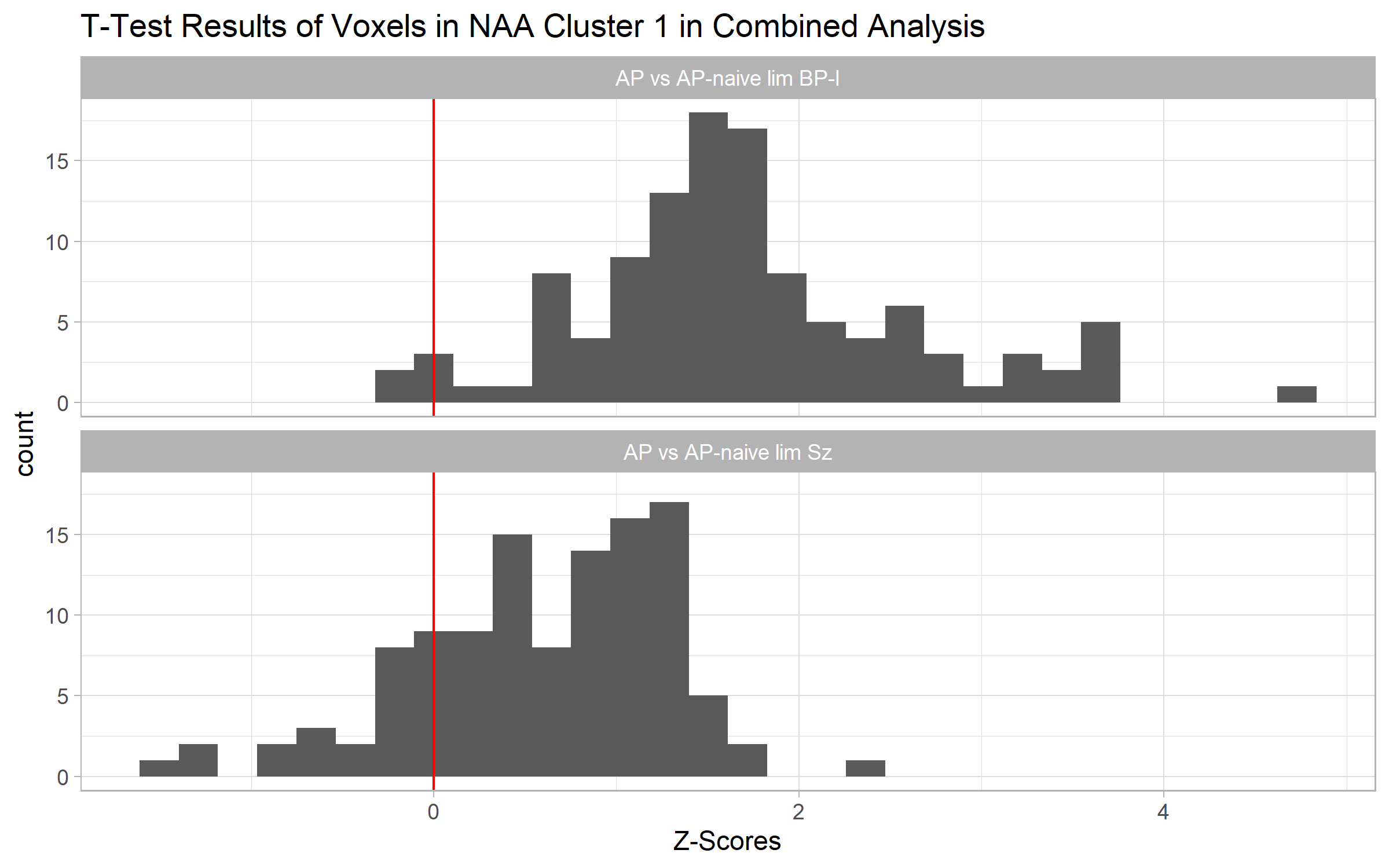
**

**Figure 2b. NAA, cluster 2: numerically higher in AP-treated than in AP-naïve in Sz and BP-I.**

**
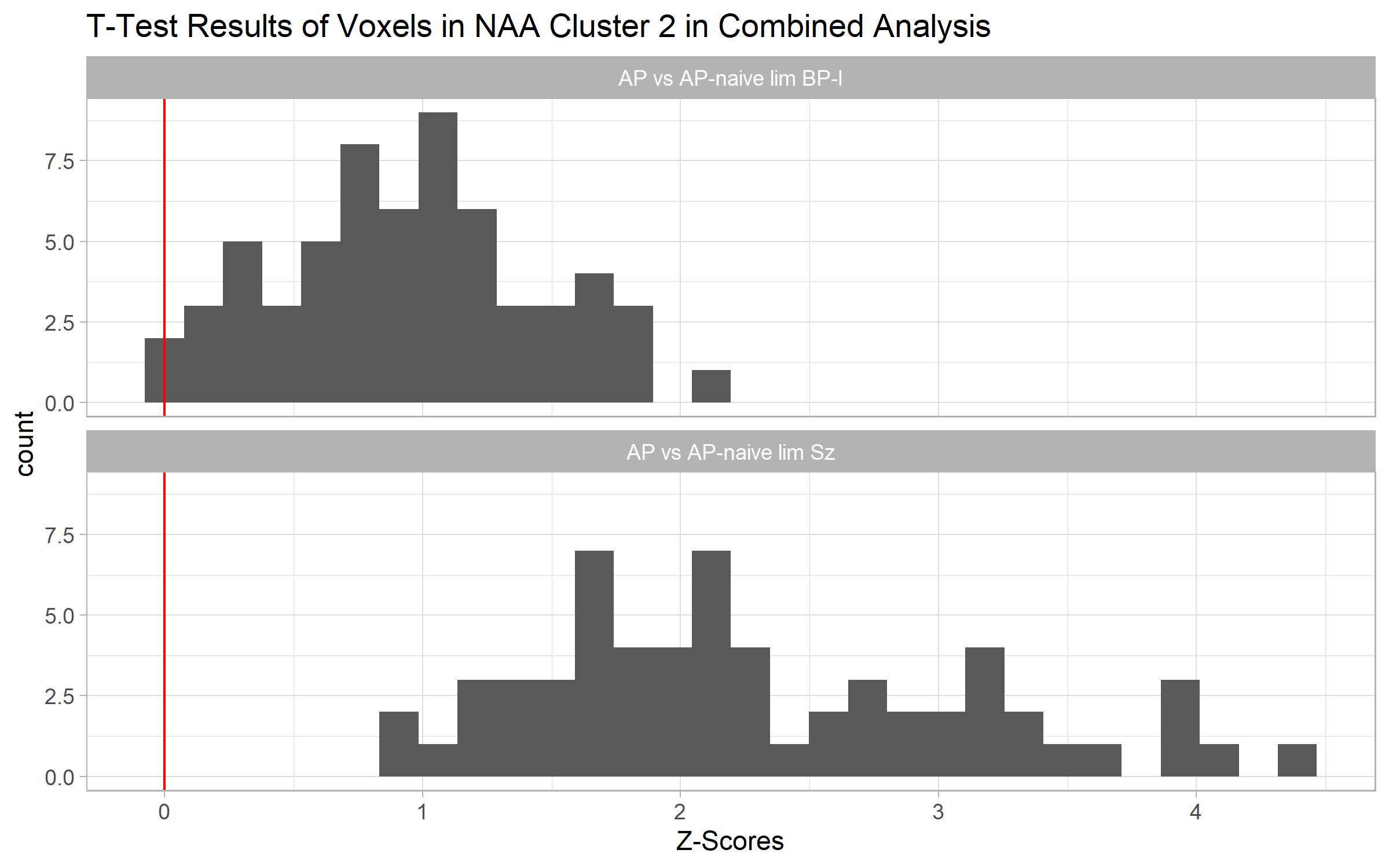
**

**Figure 2c. NAA, cluster 3: numerically higher in AP-treated than in AP-naïve in Sz and BP-I.**

**
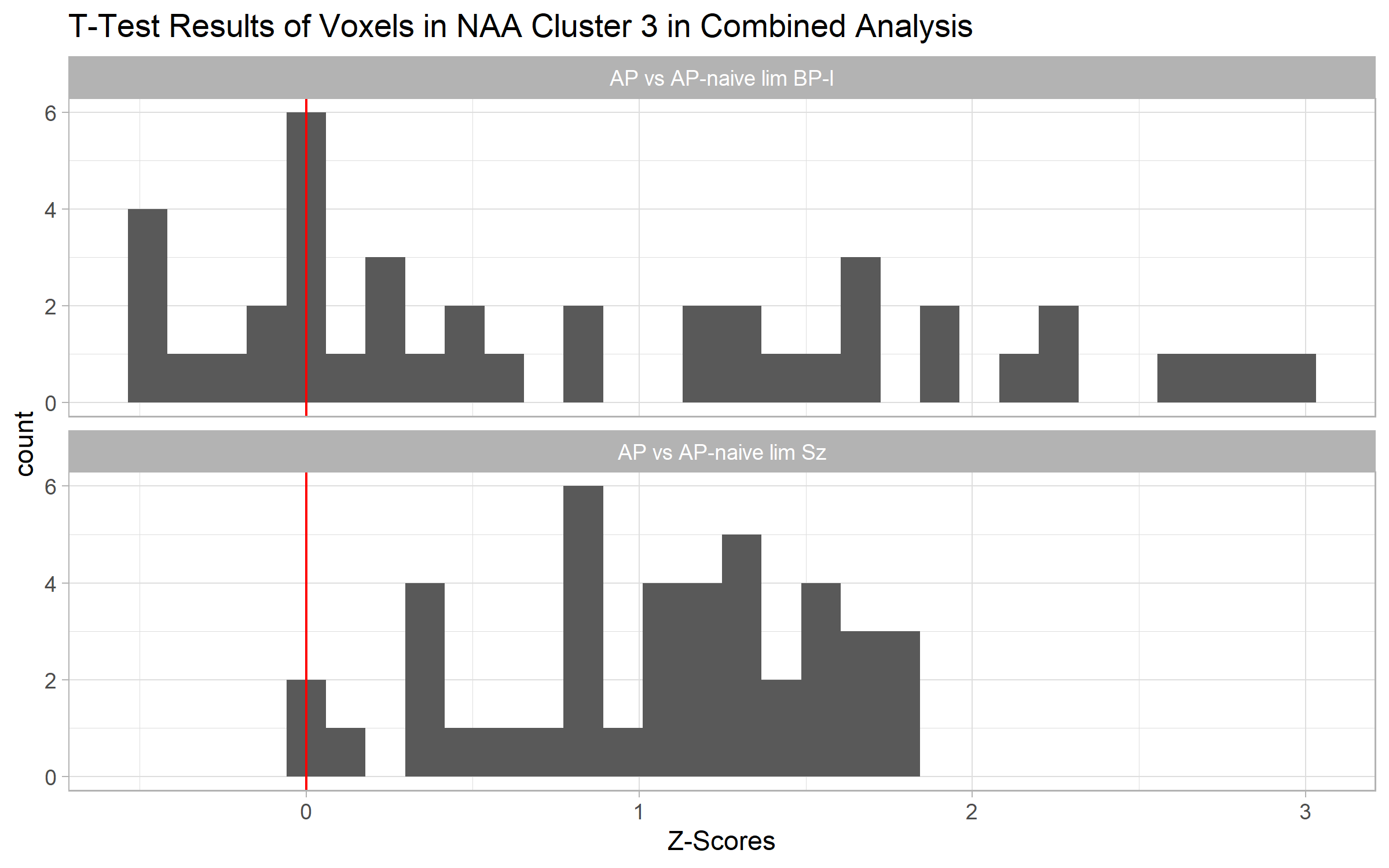
**

**Figure 3a. t-Cho, cluster 1: numerically higher in AP-treated than in AP-naïve in Sz and BP-I.**

**
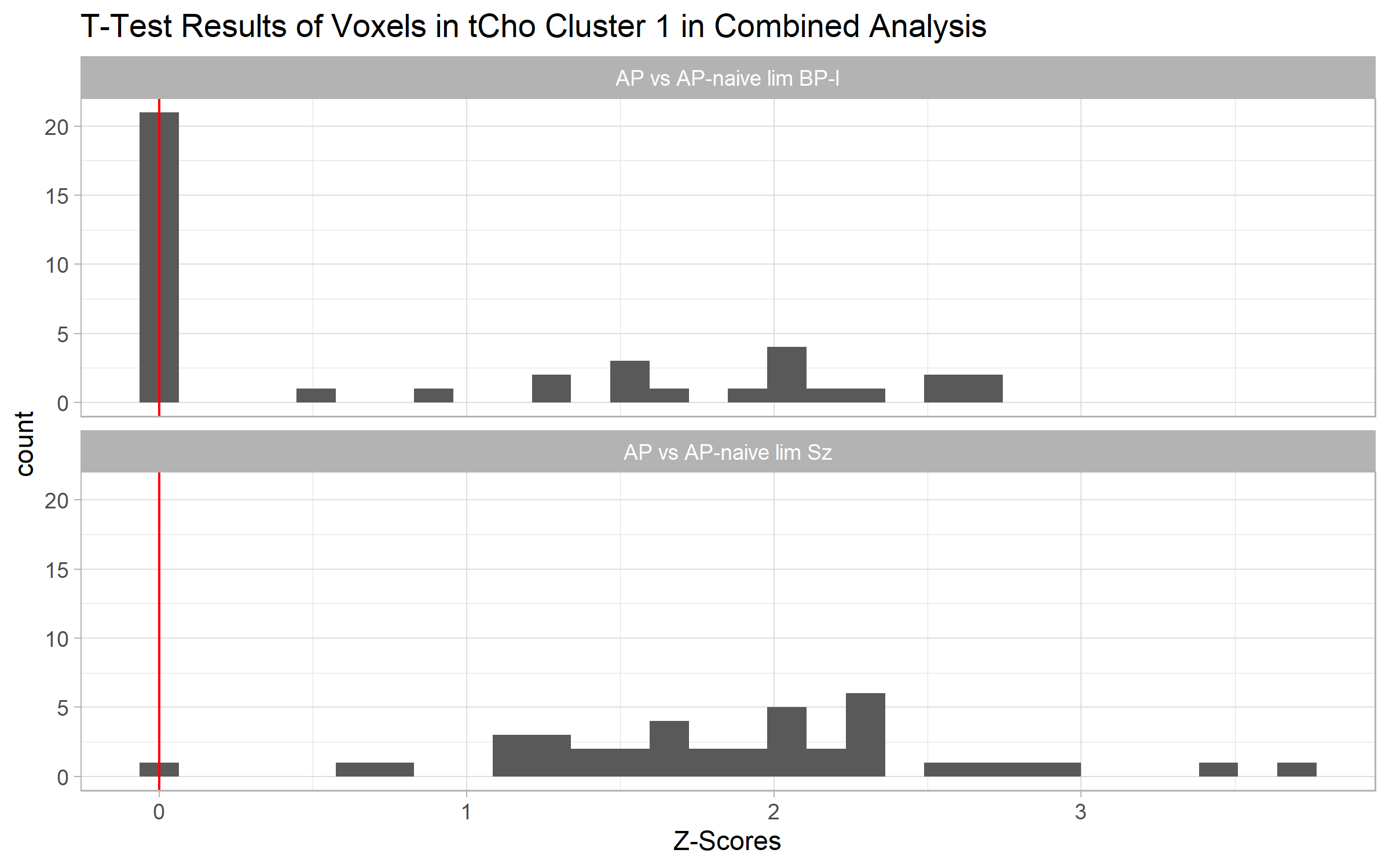
**

**Figure 3b. t-Cho, cluster 2: numerically higher in AP-treated than in AP-naïve in Sz and BP-I.**

**
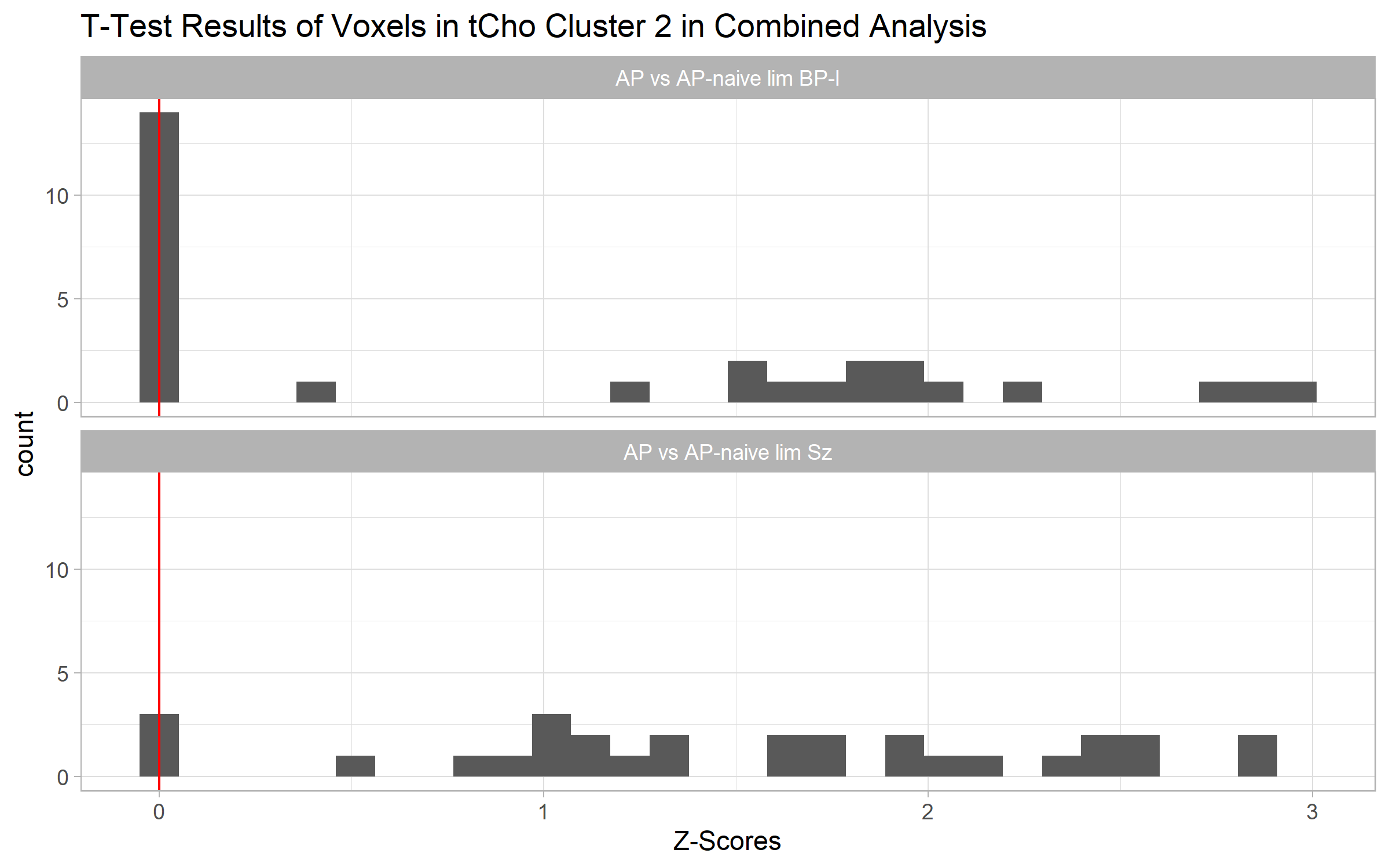
**

**Figure 4. Myo-inositol cluster: numerically higher in AP-treated than in AP-naïve in BP-I.**

**
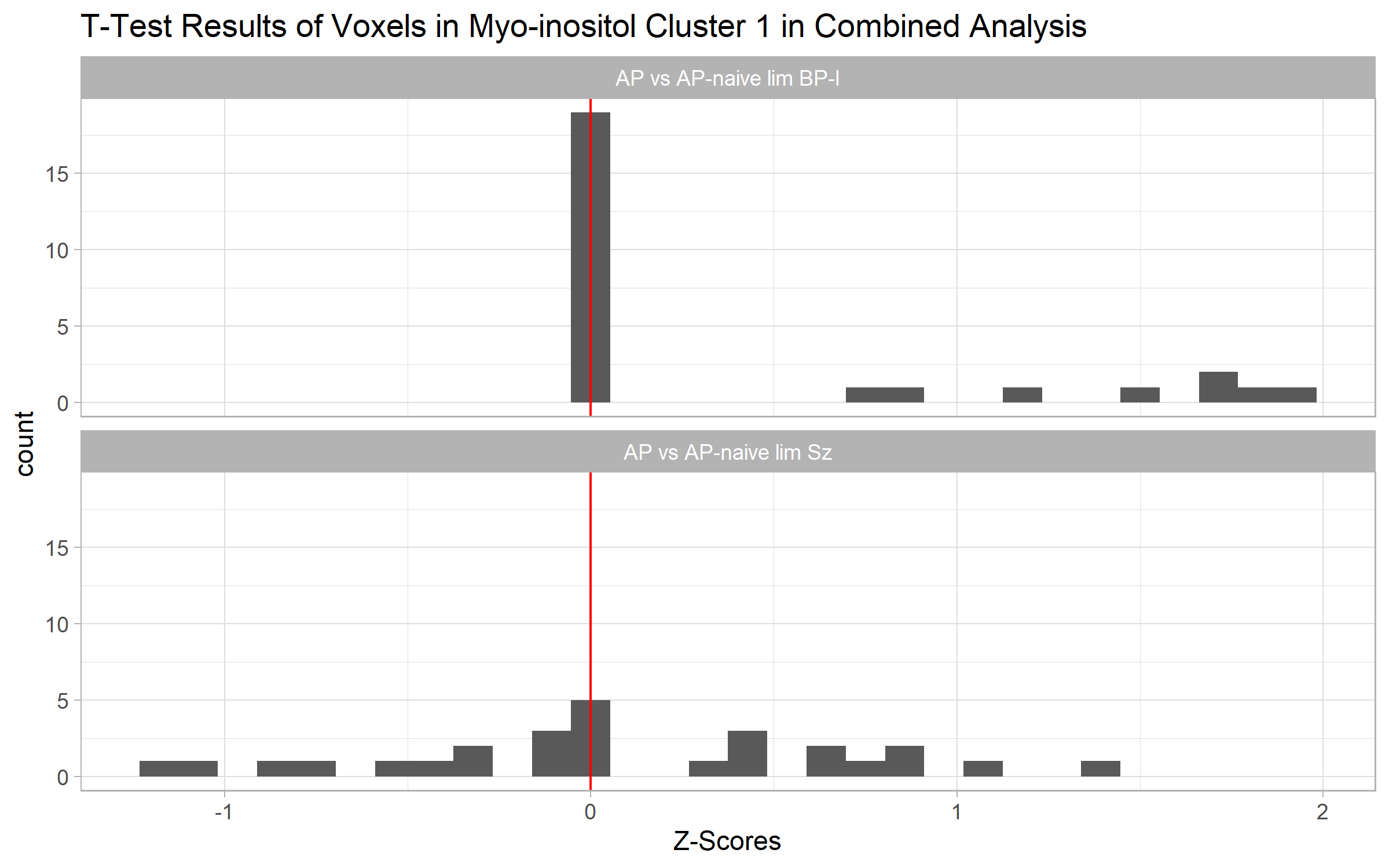
**

**Supplementary Figures (continued).**

**Figures 5 through 13:** Scatterplots of the significant correlations between symptoms or cognition (x-axis) with the weighted-average neurochemical concentrations (y-axis) in the clusters that differed between Sz and BP-I.

**Figure 5: t-Cho cluster 1 with negative symptoms.**

**
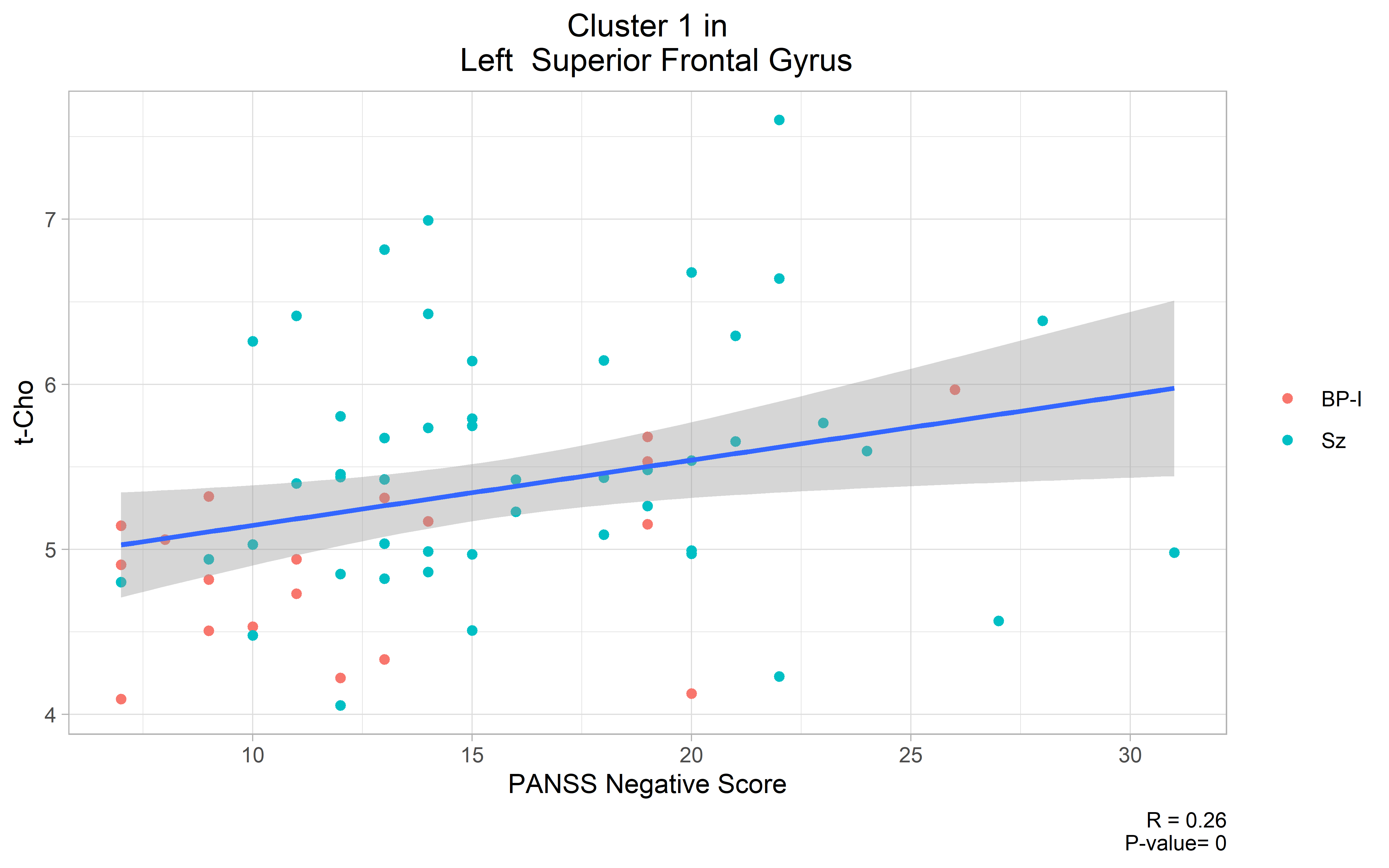
**

**Figure 6: t-Cho cluster 2 with negative symptoms.**

**
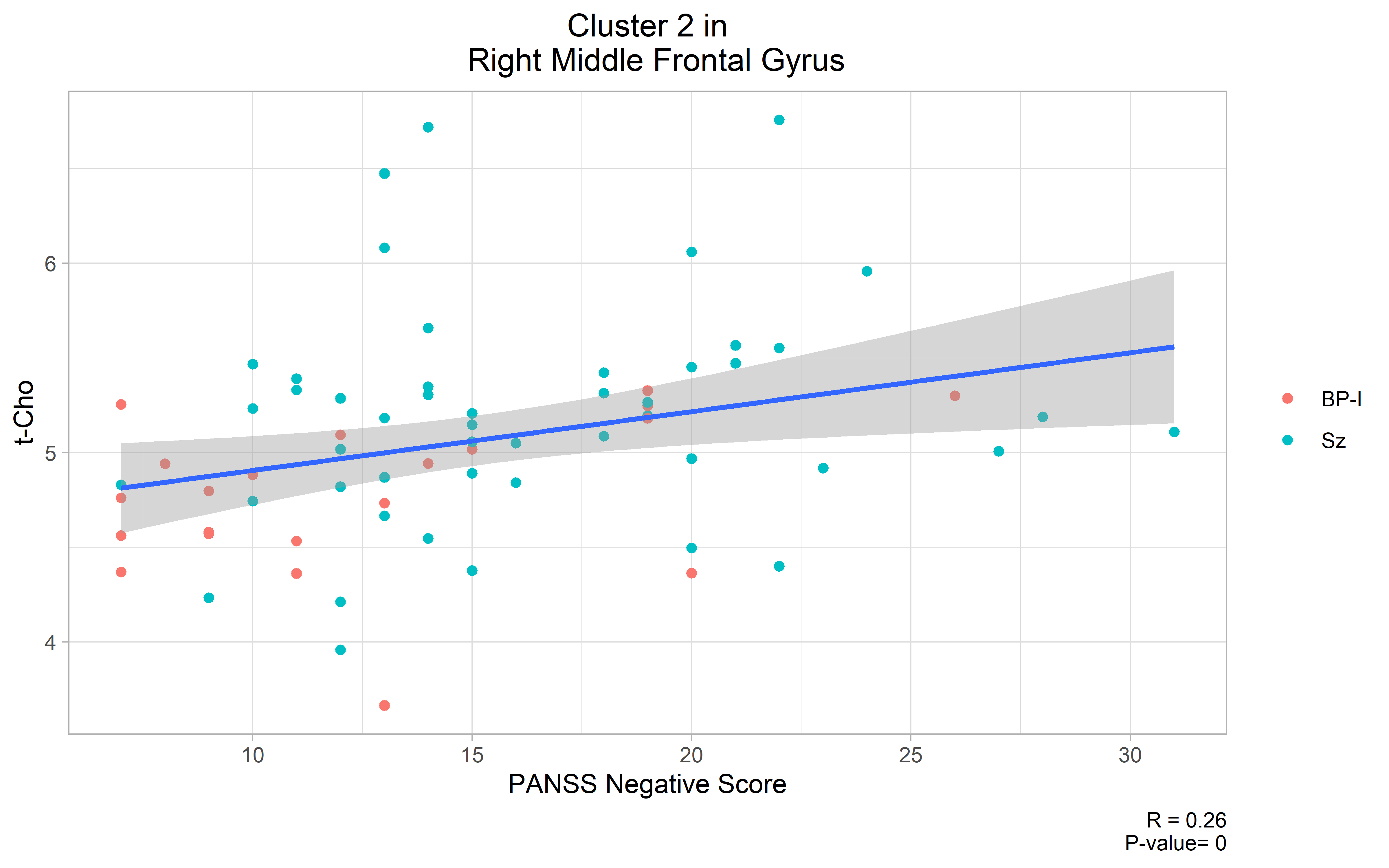
**

**Figure 7: t-Cho cluster 1 with MATRICS overall t-score.**

**
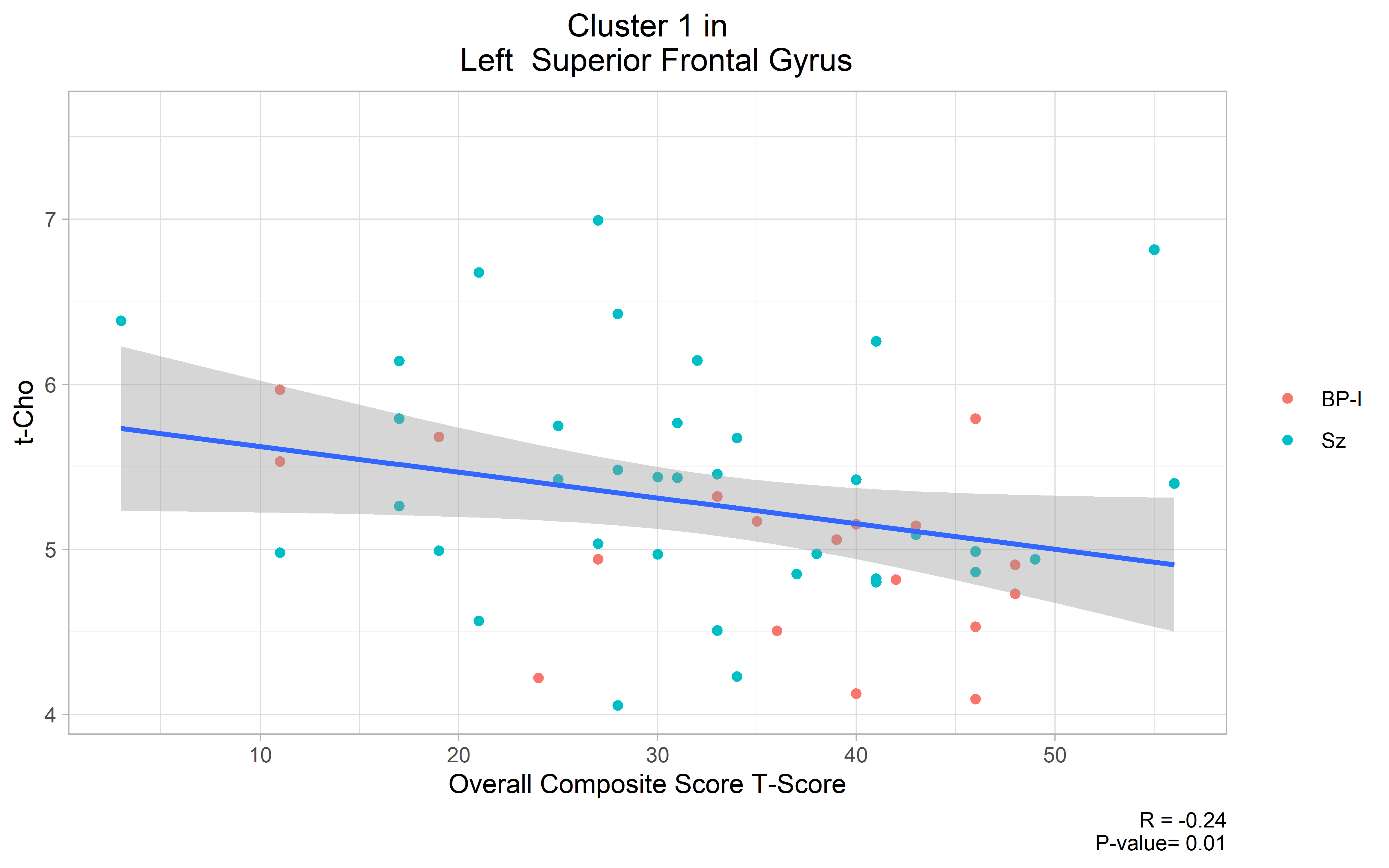
**

**Figure 8: t-Cho cluster 2 with MATRICS overall t-score.**

**
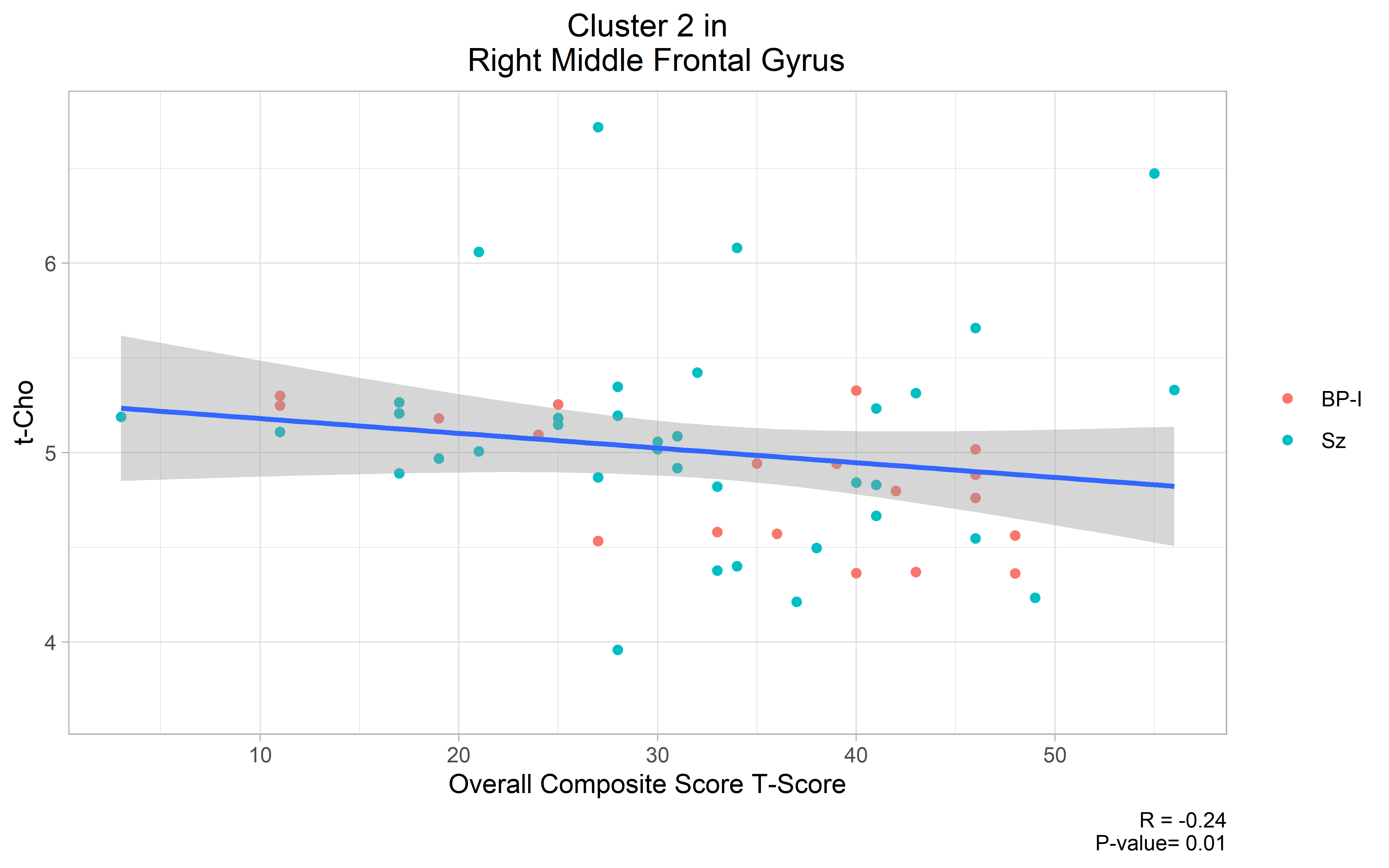
**

**Figure 9: myo-inositol cluster with negative symptoms.**

**
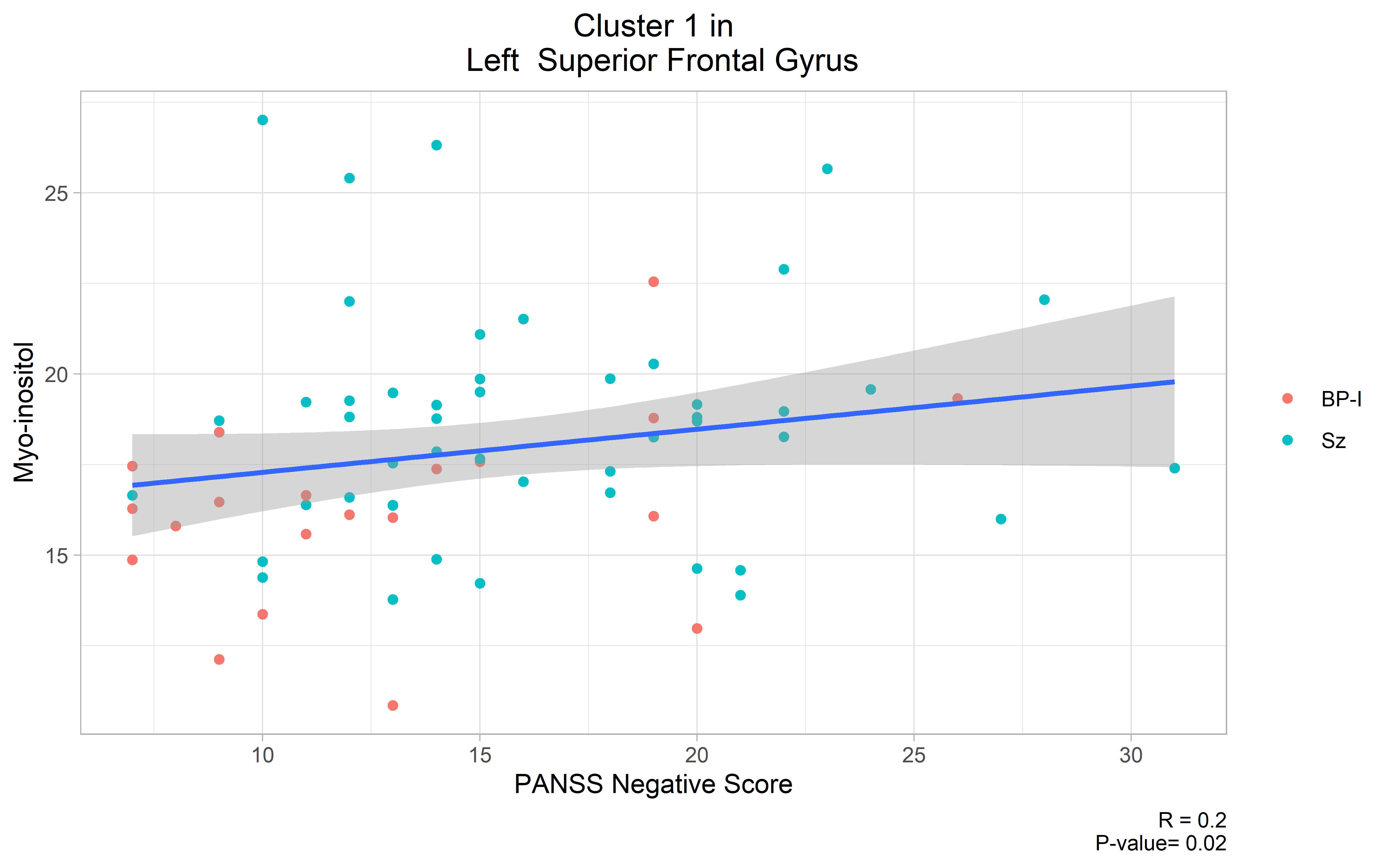
**

**Figure 10: NAA cluster 1 with negative symptoms.**

**
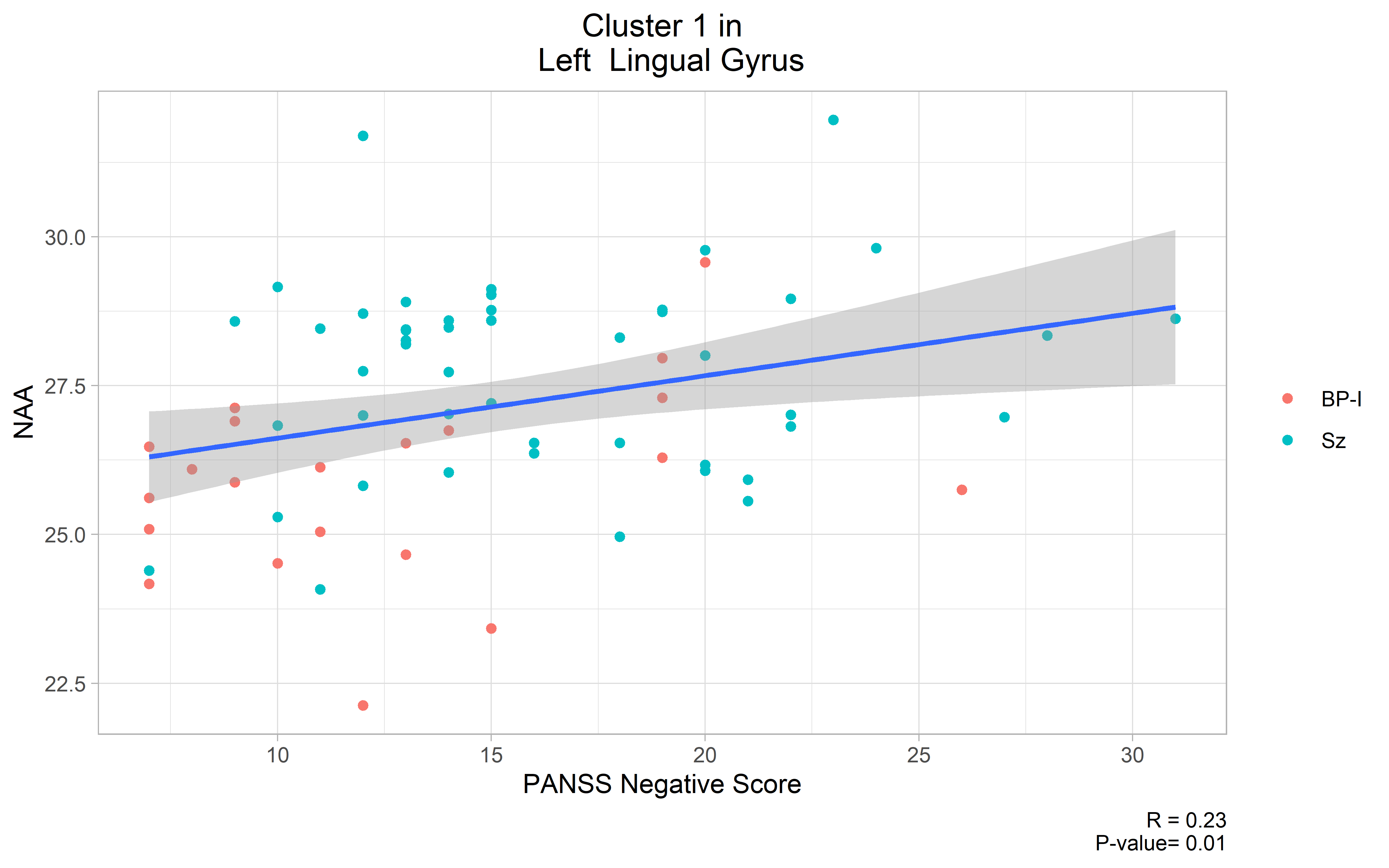
**

**Figure 11: NAA cluster 2 with negative symptoms.**

**
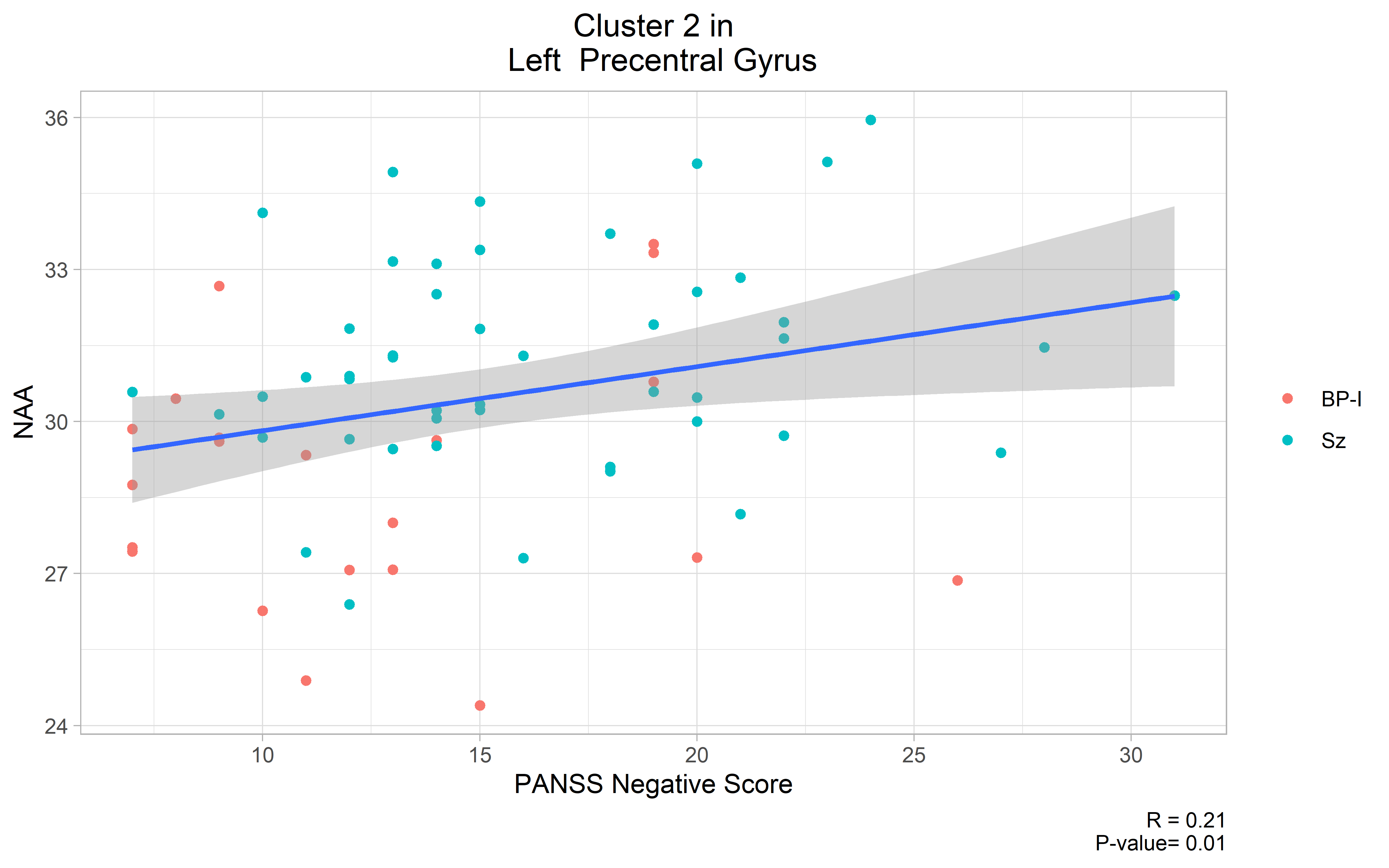
**

**Figure 12: NAA cluster 3 with negative symptoms.**

**
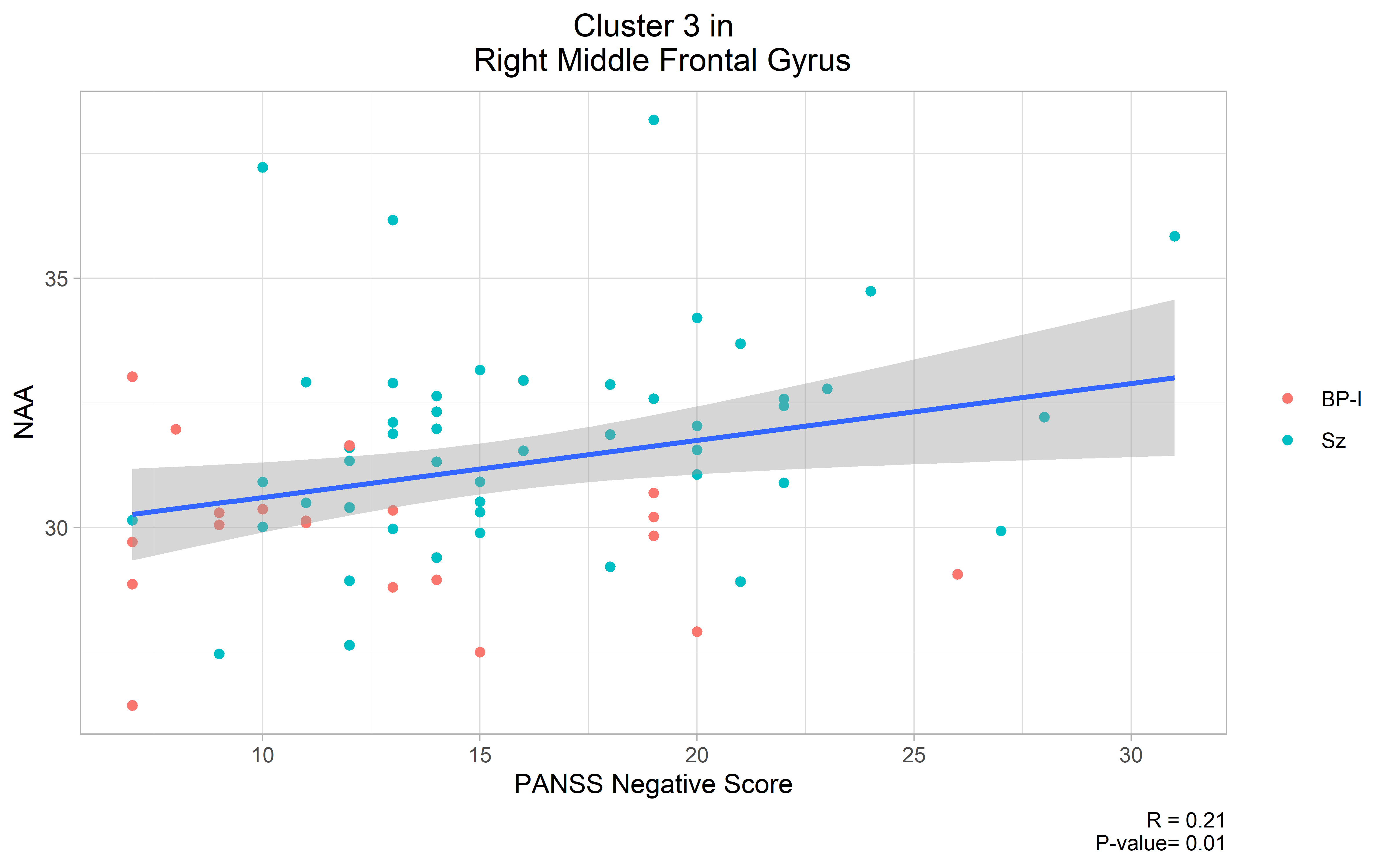
**

**Figure 13: NAA cluster 2 with MATRICS overall t-score.**

**
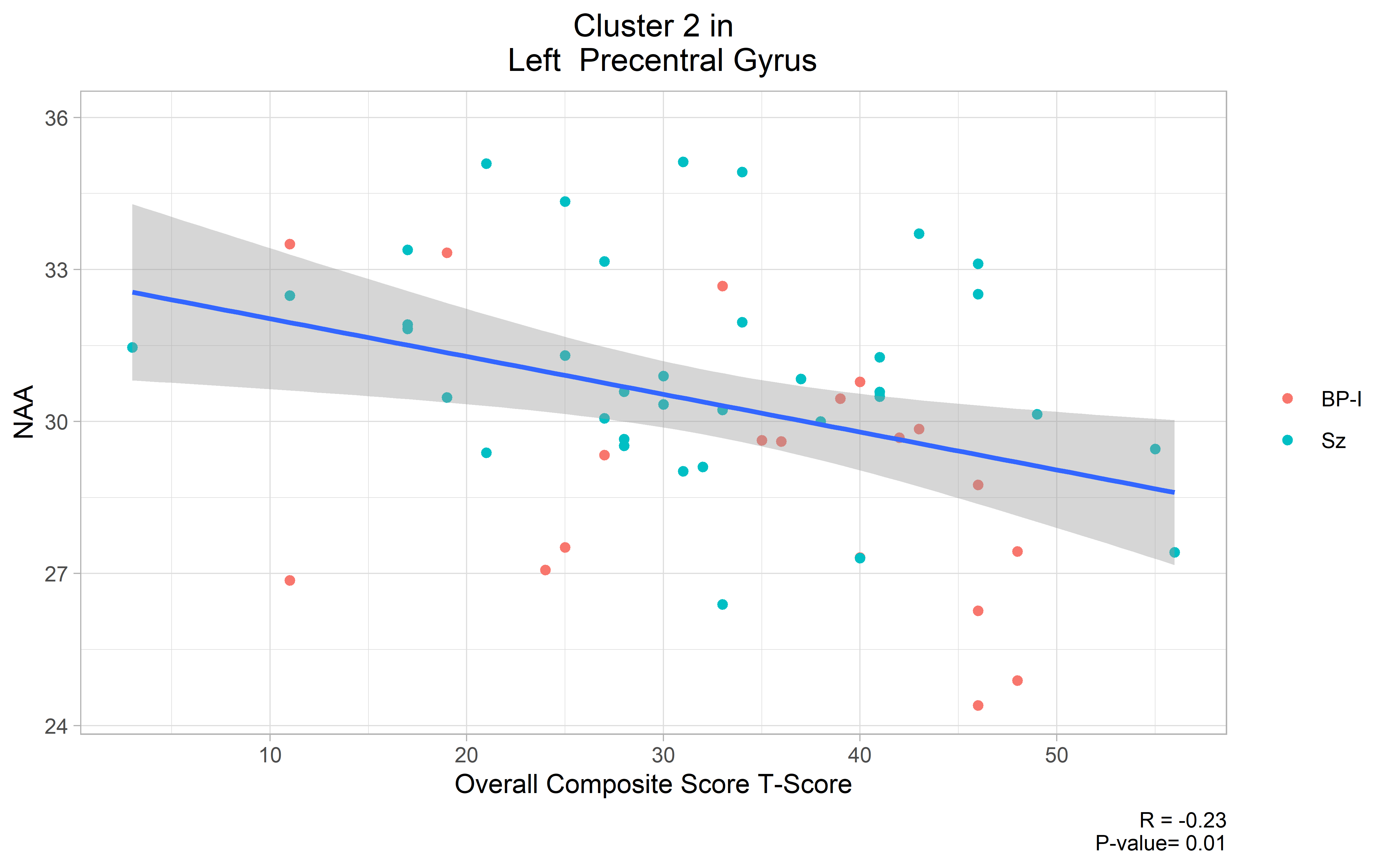
**

**Figure 14: Distribution of z-scores in the same left STG GLX group of 16 voxels in different subgroups of Schizophrenia vs Healthy Controls.**

(Only data in the first histogram represents a statistically significant cluster (Sz < HC) with most voxels with z-score between -3 and -4).

**
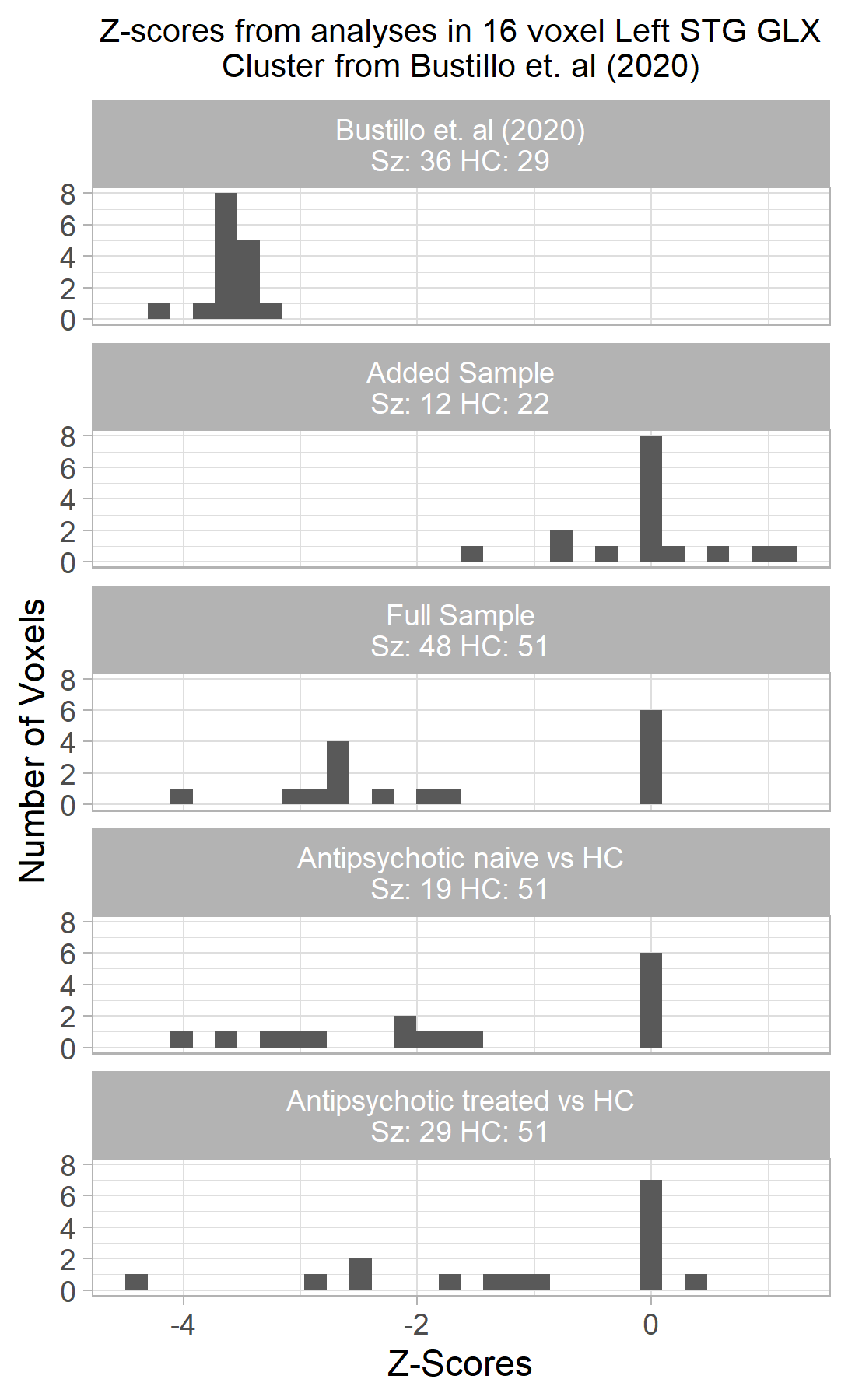
**
