# Supplementary material for: Increased Glutamate Plus Glutamine in the Right Middle Cingulate in Early Schizophrenia but Not in Bipolar Psychosis: A Whole Brain 1H-MRS Study
Source: Front Psychiatry. 2021 Jun 7;12:660850. doi: 10.3389/fpsyt.2021.660850 (PMC8215955; doi:10.3389/fpsyt.2021.660850)
Supplement: Supplementary file 2 [file Data_Sheet_2.docx]

Supplemental Tables : Anatomical labeling (per Talairach-Tournoux​ Atlas) and % of cluster accounted for metabolic group differences

| Table: 1 Schizophrenia (N=48) vs Bipolar-I (N=21) | | | | |
| --- | --- | --- | --- | --- |
| Metabolite | Group difference | CLAAV | # voxels | % location in atlas |
| Glx | Sz > BP-I | 0.05 | 19 | 60.3% Right Cingulate Gyrus.  *(60.3 % of cluster accounted for)* |
| NAA | Sz > BP-I | >0.01 | 114 | 29.9 % Left Lingual Gyrus.  13.0 % Left Posterior Cingulate   4.3 % Fusiform Gyrus.  4.1 % Left Parahippocampal Gyrus  4.0 % Left Middle Occipital Gyrus   1.9 % Left Declive  1.2 % Left Cuneus,  0.7 % Left Precuneus  0.6 % Left Inferior Occipital Gyrus  0.2 % Left Cingulate Gyrus  0.1 % Left Culmen   *( 60.0 % of cluster accounted for)* |
| NAA | Sz > BP-I | >0.01 | 61 | 34.7 % Left Precentral Gyrus  22.1 % Left Middle Frontal Gyrus  14.4 % Left Insula  3.1 % Left Inferior Frontal Gyrus  2.9 % Left Postcentral Gyrus  2.4 % Left Claustrum  0.7 % Left Lentiform Nucleus   *( 80.3 % of cluster accounted for)* |
| NAA | Sz > BP-I | 0.02 | 42 | 60.6 % Right Middle Frontal Gyrus  10.2 % Right Precentral Gyrus  5.4 % Right Superior Frontal Gyrus  0.1 % Right Insula   *(76.3 % of cluster accounted for)* |
| t-Cho | Sz > BP-I | 0.02 | 40 | 47.3 % Left Superior Frontal Gyrus  39.2 % Left Middle Frontal Gyrus  3.5 % Left Medial Frontal Gyrus  (*90.0 % of cluster accounted for).* |
| t-Cho | Sz > BP-I | 0.03 | 29 | 78.9 % Right Middle Frontal Gyrus  14.6 % Right Superior Frontal Gyrus  3.8 % Right Precentral Gyrus  *(97.3 % of cluster accounted for)* |
| Myo-inositol | Sz > BP-I | 0.03 | 27 | 65.7 % Left Superior Frontal Gyrus  23.1 % Left Middle Frontal Gyrus  5.9 % Left Medial Frontal Gyrus  (*94.7 % of cluster accounted for)* |

| Table: 2 Schizophrenia: antipsychotic-treated (med; N=29) vs Schizophrenia antipsychotic-naive (N=19) | | | | |
| --- | --- | --- | --- | --- |
| Metabolite | Group difference | CLAAV | # voxels | % location in atlas |
| t-Cr | Sz_med_> Sz_naive_ | 0.02 | 34 | 25.0 % Left Parahippocampal Gyrus  10.5 % Left Culmen  4.3 % Left Insula  4.0 % Left Lentiform Nucleus  2.6 % Left Claustrum  0.8 % Left Caudate  0.6 % Left Superior Temporal Gyrus  *(47.9 % of cluster accounted for)* |
| t-Cr | Sz_med_> Sz_naive_ | 0.03 | 27 | 51.9 % Right Declive  16.5 % Right Fusiform Gyrus  16.1 % Right Uvula  7.4 % Right Culmen  6.0 % Right Tuber  0.9 % Right Inferior Occipital Gyrus  0.9 % Right Middle Occipital Gyrus  0.2 % Right Lingual Gyrus  *(100.0% of cluster accounted for)* |
| t-Cho | Sz_med_> Sz_naive_ | 0.02 | 28 | 35.6 % Right Declive  19.6 % Right Pyramis  16.3 % Right Tuber  11.0 % Right Uvula  8.6 % Right Cerebellar Tonsil  5.7 % Right Fusiform Gyrus  2.1 % Right Culmen  *(98.8 % of cluster accounted for)* |
| t-Cho | Sz_med_> Sz_naive_ | 0.03 | 23 | 49.7 % Left Middle Frontal Gyrus  40.0 % Left Inferior Frontal Gyrus  5.8 % Left Precentral Gyrus  *(95.5 % of cluster accounted for)* |
| t-Cho | Sz_med_> Sz_naive_ | 0.03 | 18 | 21.7 % Left Insula  7.4 % Left Claustrum  7.3 % Left Lentiform Nucleus  1.1 % Left Superior Temporal Gyrus  0.6 % Left Caudate  *(38.2 % of cluster accounted for)* |
| t-Cho | Sz_med_> Sz_naive_ | 0.04 | 16 | 46.9 % Left Parahippocampal Gyrus  38.4 % Left Culmen  (85.3 % of cluster accounted for) |
| NAA | Sz_med_> Sz_naive_ | 0.04 | 21 | 61.5 % Left Middle Frontal Gyrus  15.7 % Left Precentral Gyrus  *(77.2 % of cluster accounted for)* |

| Table: 3 Spectral quality and gray matter fraction in metabolite clusters that differ between schizophrenia and bipolar-I disorders | | | | | | | |
| --- | --- | --- | --- | --- | --- | --- | --- |
| Metabolite | Cluster | Metric | Sz Mean | Sz SD | BP-I Mean | BP-I SD | Mann Whitney*  P-Value |
| t-Cho | 1 | Linewidth | 6.69 | 0.82 | 6.48 | 0.77 | 0.26 |
| t-Cho | 1 | CRLB | 8.71 | 2.57 | 9.23 | 3.08 | 0.75 |
| t-Cho | 1 | GM_Fraction^~^ | 0.50 | 0.06 | 0.51 | 0.06 | 0.87 |
| t-Cho | 2 | Linewidth | 6.15 | 0.88 | 5.96 | 0.70 | 0.33 |
| t-Cho | 2 | CRLB | 7.43 | 2.51 | 8.16 | 2.79 | 0.24 |
| t-Cho | 2 | GM_Fraction | 0.57 | 0.07 | 0.60 | 0.04 | 0.07 |
| Glx | 1 | Linewidth | 6.07 | 0.60 | 6.23 | 1.01 | 0.59 |
| Glx | 1 | CRLB | 10.96 | 1.95 | 13.23 | 2.39 | 0.00 |
| Glx | 1 | GM_Fraction | 0.10 | 0.05 | 0.08 | 0.04 | 0.09 |
| NAA | 1 | Linewidth | 6.57 | 0.73 | 6.45 | 0.71 | 0.52 |
| NAA | 1 | CRLB | 4.22 | 0.38 | 4.61 | 0.93 | 0.06 |
| NAA | 1 | GM_Fraction | 0.28 | 0.04 | 0.27 | 0.04 | 0.27 |
| NAA | 2 | Linewidth | 6.41 | 0.78 | 6.42 | 1.00 | 0.83 |
| NAA | 2 | CRLB | 4.55 | 0.99 | 5.11 | 1.26 | 0.03 |
| NAA | 2 | GM_Fraction | 0.44 | 0.05 | 0.42 | 0.04 | 0.06 |
| NAA | 3 | Linewidth | 6.22 | 0.72 | 6.03 | 0.68 | 0.27 |
| NAA | 3 | CRLB | 4.57 | 1.25 | 5.13 | 2.33 | 0.31 |
| NAA | 3 | GM_Fraction | 0.44 | 0.07 | 0.45 | 0.06 | 0.41 |
| Myo-inositol | 1 | Linewidth | 7.23 | 1.07 | 6.97 | 0.89 | 0.30 |
| Myo-inositol | 1 | CRLB | 9.95 | 2.82 | 10.98 | 2.86 | 0.23 |
| Myo-inositol | 1 | GM_Fraction | 0.48 | 0.06 | 0.46 | 0.07 | 0.36 |
| * Mann Whitney used because of data skewing.  ^~^GM_F= GM/GM+WM in voxel | | | | | | | |

| Table 4. No significant differences in number of voxels with best, intermediate or imputed spectra in schizophrenia vs. bipolar-1 groups for the five metabolite masks | | | | |
| --- | --- | --- | --- | --- |
| Voxel type | Group | Metabolite | Mean | SD |
| Intermediate | BP-I | t-Cho | 167.86 | 80.19 |
| Intermediate | SZ | t-Cho | 144.40 | 34.88 |
| Imputed | BP-I | t-Cho | 1384.29 | 477.12 |
| Imputed | SZ | t-Cho | 1217.42 | 231.37 |
| Best | BP-I | t-Cho | 9301.43 | 781.77 |
| Best | SZ | t-Cho | 9120.29 | 768.65 |
| Brain | BP-I | t-Cho | 10685.71 | 488.82 |
| Brain | SZ | t-Cho | 10337.71 | 712.01 |
| Intermediate | BP-I | t-Cr | 151.05 | 63.12 |
| Intermediate | SZ | t-Cr | 134.73 | 26.25 |
| Imputed | BP-I | t-Cr | 1275.52 | 354.88 |
| Imputed | SZ | t-Cr | 1139.73 | 205.06 |
| Best | BP-I | t-Cr | 9428.10 | 670.90 |
| Best | SZ | t-Cr | 9206.40 | 731.87 |
| Brain | BP-I | t-Cr | 10703.62 | 474.34 |
| Brain | SZ | t-Cr | 10346.13 | 706.33 |
| Intermediate | BP-I | GLX | 405.90 | 126.81 |
| Intermediate | SZ | GLX | 391.90 | 101.48 |
| Imputed | BP-I | GLX | 1924.00 | 616.32 |
| Imputed | SZ | GLX | 1774.31 | 344.07 |
| Best | BP-I | GLX | 8640.14 | 1031.68 |
| Best | SZ | GLX | 8411.98 | 903.96 |
| Brain | BP-I | GLX | 10564.14 | 542.50 |
| Brain | SZ | GLX | 10186.29 | 778.26 |
| Intermediate | BP-I | Myo-inositol | 254.38 | 116.64 |
| Intermediate | SZ | Myo-inositol | 240.83 | 71.02 |
| Imputed | BP-I | Myo-inositol | 1587.29 | 519.40 |
| Imputed | SZ | Myo-inositol | 1433.38 | 265.86 |
| Best | BP-I | Myo-inositol | 8945.00 | 921.47 |
| Best | SZ | Myo-inositol | 8691.98 | 914.36 |
| Brain | BP-I | Myo-inositol | 10532.29 | 540.22 |
| Brain | SZ | Myo-inositol | 10125.35 | 839.37 |
| Intermediate | BP-I | NAA | 141.76 | 66.08 |
| Intermediate | SZ | NAA | 123.75 | 27.45 |
| Imputed | BP-I | NAA | 1275.71 | 354.80 |
| Imputed | SZ | NAA | 1159.67 | 210.99 |
| Best | BP-I | NAA | 9412.33 | 667.03 |
| Best | SZ | NAA | 9178.42 | 739.94 |
| Brain | BP-I | NAA | 10688.05 | 481.91 |
| Brain | SZ | NAA | 10338.08 | 705.40 |
| ***Best:*** *spectra with overall line widths= 2-12 Hz; specific spectral fits for each metabolite of Cramér– Rao lower bound (CRLB)= 1-20%; and voxel CSF fraction <30%*  ***Intermediate****: spectra with* *linewidths greater than 1 and less than or equal to 16; CRLB= 1-99%; f_CSF_≤0.3; and at least 18 of the 26 nearest neighboring voxels are best spectra.*  ***Brain:*** *# of voxels with best plus intermediate spectral*  ***Imputed****: voxels with poor spectra in individual subjects (non-best or intermediate) but for which >75% of the group sample has best or intermediate spectra, were imputed using the diagnostic group (Sz, BP-I, HC) mean concentration for that voxel.* | | | | |

| Table 5: Demographic and clinical characteristics of  originally reported (Bustillo et. al. 2020) and added schizophrenia subjects | | | |
| --- | --- | --- | --- |
|  | **Added Sz Subjects**  **(N=16)** | **Original Sz Subjects (N=32)** | **p value** |
| **RACE** |  |  | 0.503 |
| N-Miss | 0 | 1 |  |
| African American | 1 (6.2%) | 3 (9.7%) |  |
| Asian | 1 (6.2%) | 0 (0.0%) |  |
| Native American | 1 (6.2%) | 1 (3.2%) |  |
| White | 13 (81.2%) | 27 (87.1%) |  |
| **Vascular risk score** |  |  | 1.000 |
| 0 | 15 (93.8%) | 30 (93.8%) |  |
| 1 | 1 (6.2%) | 2 (6.2%) |  |
| **Alcohol** |  |  | 1.000 |
| No | 14 (87.5%) | 28 (87.5%) |  |
| Yes | 2 (12.5%) | 4 (12.5%) |  |
| **Sedative** |  |  | 1.0 |
| No | 16 (100.0%) | 32 (100.0%) |  |
| **Cannabis** |  |  | 0.838 |
| No | 8 (50.0%) | 15 (46.9%) |  |
| Yes | 8 (50.0%) | 17 (53.1%) |  |
| **Stimulants** |  |  | 0.307 |
| No | 16 (100.0%) | 30 (93.8%) |  |
| Yes | 0 (0.0%) | 2 (6.2%) |  |
| **Opioids** |  |  | 0.475 |
| No | 16 (100.0%) | 31 (96.9%) |  |
| Yes | 0 (0.0%) | 1 (3.1%) |  |
| **PCP** |  |  | 0.307 |
| No | 16 (100.0%) | 30 (93.8%) |  |
| Yes | 0 (0.0%) | 2 (6.2%) |  |
| **Hallucinogens** |  |  | 0.460 |
| No | 14 (87.5%) | 30 (93.8%) |  |
| Yes | 2 (12.5%) | 2 (6.2%) |  |
| **Inhalants** |  |  | 0.206 |
| No | 16 (100.0%) | 29 (90.6%) |  |
| Yes | 0 (0.0%) | 3 (9.4%) |  |
| **SES** |  |  | 0.016 |
| N-Miss | 0 | 1 |  |
| Mean (SD) | 6.875 (0.806) | 5.839 (1.551) |  |
| Range | 4.000 - 8.000 | 1.000 - 7.000 |  |
| **Familial SES** |  |  | 0.395 |
| N-Miss | 0 | 2 |  |
| Mean (SD) | 3.875 (1.821) | 4.367 (1.866) |  |
| Range | 2.000 - 7.000 | 1.000 - 7.000 |  |
| **Tardive dyskinesia** |  |  | 0.160 |
| Mean (SD) | 7.000 (0.000) | 7.000 (0.000) |  |
| Range | 7.000 - 7.000 | 7.000 - 7.000 |  |
| **Akathisia** |  |  | 0.173 |
| Mean (SD) | 0.062 (0.250) | 0.500 (1.244) |  |
| Range | 0.000 - 1.000 | 0.000 - 5.000 |  |
| **Parkinsonism** |  |  | 0.787 |
| Mean (SD) | 8.125 (0.500) | 8.094 (0.296) |  |
| Range | 8.000 - 10.000 | 8.000 - 9.000 |  |
| **Depression** |  |  | 0.876 |
| Mean (SD) | 2.938 (4.768) | 2.750 (3.417) |  |
| Range | 0.000 - 16.000 | 0.000 - 14.000 |  |
| **Matrics Overall T Score** |  |  | 0.130 |
| N-Miss | 0 | 12 |  |
| Mean (SD) | 28.188 (12.018) | 34.200 (11.153) |  |
| Range | 3.000 - 49.000 | 17.000 - 56.000 |  |
| **Mania** |  |  | 0.414 |
| Mean (SD) | 2.812 (4.430) | 1.844 (3.511) |  |
| Range | 0.000 - 14.000 | 0.000 - 12.000 |  |
| **Positive symptoms** |  |  | 0.200 |
| Mean (SD) | 14.625 (5.414) | 16.719 (5.176) |  |
| Range | 7.000 - 23.000 | 8.000 - 29.000 |  |
| **Negative symptoms** |  |  | 0.526 |
| Mean (SD) | 17.000 (6.088) | 15.969 (4.823) |  |
| Range | 9.000 - 31.000 | 7.000 - 27.000 |  |
| **Age** |  |  | 0.276 |
| Mean (SD) | 21.500 (3.706) | 22.812 (3.971) |  |
| Range | 17.000 - 30.000 | 17.000 - 31.000 |  |
| **Psychosis onset (yr)** |  |  | 0.942 |
| Mean (SD) | 20.214 (3.536) | 20.312 (4.802) |  |
| Range | 15.000 - 26.750 | 7.000 - 31.000 |  |
| Range | 16.000 - 26.750 | 13.000 - 30.000 |  |
| **Antipsychotic use** |  |  | 0.007 |
| No | 2 (12.5%) | 17 (53.1%) |  |
| Yes | 14 (87.5%) | 15 (46.9%) |  |
| **Antipsychotic dose** |  |  | 0.032 |
| Mean (SD) | 9.322 (6.765) | 4.803 (6.622) |  |
| Range | 0.000 - 25.400 | 0.000 - 27.000 |  |
| **Gender** |  |  | 0.653 |
| female | 4 (25.0%) | 10 (31.2%) |  |
| male | 12 (75.0%) | 22 (68.8%) |  |
| **Smoker** |  |  | 1.000 |
| No | 13 (81.2%) | 26 (81.2%) |  |
| Yes | 3 (18.8%) | 6 (18.8%) |  |

| Table 6: Demographic and clinical characteristics of  originally reported (Bustillo et. al. 2020) and added healthy control subjects | | | |
| --- | --- | --- | --- |
|  | **Added HC Subjects**  **(N=22)** | **Original HC Subjects (N=29)** | **p value** |
| **RACE** |  |  | 0.143 |
| African American | 2 (9.1%) | 1 (3.4%) |  |
| Asian | 3 (13.6%) | 0 (0.0%) |  |
| Native American | 2 (9.1%) | 2 (6.9%) |  |
| White | 15 (68.2%) | 26 (89.7%) |  |
| **Vascular risk score** |  |  | 0.379 |
| 0 | 22 (100.0%) | 28 (96.6%) |  |
| 1 | 0 (0.0%) | 1 (3.4%) |  |
| **Alcohol** |  |  | 0.086 |
| N-Miss | 1 | 13 |  |
| No | 15 (71.4%) | 15 (93.8%) |  |
| Yes | 6 (28.6%) | 1 (6.2%) |  |
| **Sedative** |  |  | 0.411 |
| N-Miss | 1 | 13 |  |
| No | 21 (100.0%) | 16 (100.0%) |  |
| **Cannabis** |  |  | 0.718 |
| N-Miss | 1 | 13 |  |
| No | 19 (90.5%) | 15 (93.8%) |  |
| Yes | 2 (9.5%) | 1 (6.2%) |  |
| **Stimulants** |  |  | 1.0 |
| N-Miss | 1 | 13 |  |
| No | 21 (100.0%) | 16 (100.0%) |  |
| Yes | 0 (0.0%) | 0 (0.0%) |  |
| **Opioids** |  |  | 1.0 |
| N-Miss | 1 | 13 |  |
| No | 21 (100.0%) | 16 (100.0%) |  |
| Yes | 0 (0.0%) | 0 (0.0%) |  |
| **PCP** |  |  | 1.0 |
| N-Miss | 1 | 13 |  |
| No | 21 (100.0%) | 16 (100.0%) |  |
| Yes | 0 (0.0%) | 0 (0.0%) |  |
| **Hallucinogens** |  |  | 1.0 |
| N-Miss | 1 | 13 |  |
| No | 21 (100.0%) | 16 (100.0%) |  |
| Yes | 0 (0.0%) | 0 (0.0%) |  |
| **Inhalants** |  |  | 1.0 |
| N-Miss | 1 | 13 |  |
| No | 21 (100.0%) | 16 (100.0%) |  |
| Yes | 0 (0.0%) | 0 (0.0%) |  |
| **SES** |  |  | 0.164 |
| Mean (SD) | 4.636 (1.590) | 4.069 (1.280) |  |
| Range | 2.000 - 7.000 | 1.000 - 7.000 |  |
| **Family SES** |  |  | 0.046 |
| Mean (SD) | 3.909 (1.823) | 2.931 (1.580) |  |
| Range | 1.000 - 7.000 | 1.000 - 7.000 |  |
| **MATRICS Overall T Score** |  |  | 0.924 |
| N-Miss | 0 | 5 |  |
| Mean (SD) | 48.045 (8.471) | 48.250 (5.810) |  |
| Range | 25.000 - 63.000 | 38.000 - 59.000 |  |
| **Age** |  |  | 0.116 |
| Mean (SD) | 24.773 (4.418) | 22.897 (3.931) |  |
| Range | 14.000 - 30.000 | 17.000 - 35.000 |  |
| **Gender** |  |  | 0.771 |
| female | 10 (45.5%) | 12 (41.4%) |  |
| male | 12 (54.5%) | 17 (58.6%) |  |
| **Smoker** |  |  | 0.023 |
| 0 | 22 (100.0%) | 23 (79.3%) |  |
| 1 | 0 (0.0%) | 6 (20.7%) |  |
